# Masting Breakdown in European Beech Reduces Fitness Benefits of Masting, Partly Explained by Climate Change

**DOI:** 10.1002/ece3.73809

**Published:** 2026-06-08

**Authors:** Cherine C. Jantzen, Joseph B. Burant, Marlène Gamelon, Elisabeth S. Bakker, Marcel E. Visser

**Affiliations:** ^1^ Department of Animal Ecology Netherlands Institute of Ecology (NIOO‐KNAW) Wageningen the Netherlands; ^2^ Groningen Institute for Evolutionary Life Sciences (GELIFES), University of Groningen Groningen the Netherlands; ^3^ Université Claude Bernard Lyon 1, CNRS, VetAgro Sup, UMR 5558 Biométrie et Biologie Evolutive Villeurbanne Cedex France; ^4^ Department of Terrestrial Ecology Netherlands Institute of Ecology (NIOO‐KNAW) Wageningen the Netherlands; ^5^ Wildlife Ecology and Conservation Group Wageningen University & Research Wageningen the Netherlands

**Keywords:** climate change, *Fagus sylvatica*, mast seeding, pollen efficiency, predator satiation, reproductive ecology, synchrony, weather cues

## Abstract

Masting, highly synchronised but temporally variable seed production, is initiated by weather cues and is thus highly sensitive to climate change. Changes in these cues can lead to a masting breakdown, reducing the fitness benefits of masting through decreasing pollination efficiency and increasing predation risk for seeds. Here, we use 50 years of individual tree data on annual seed production of European beech ( *Fagus sylvatica* ) in the Netherlands to assess temporal changes in masting patterns and their consequences for the selective benefits of masting. Additionally, we use a novel approach to identify which weather cues initiate reproduction, assess their temporal changes, and test whether they account for the observed changes in masting. We show that synchrony and inter‐annual variation in beechnut production have declined, resulting in a masting breakdown in the late‐2000s, since which there has been constant, but low, seed production each year. Consequently, predation risk increased almost three‐fold, while pollination became less efficient, together reducing the fitness benefits of masting. Seed production was driven by precipitation and temperatures in the year of seed fall and the two preceding years, but the periods within the year in which trees respond to each climate variable differ in both timing and duration. Interestingly, only temperature, not precipitation, has changed over time, but this change only partly explained the observed changes in masting patterns. Masting breakdown is shown across the species range, but its fitness consequences remain understudied, because detailed, individual‐level, long‐term data are required but still rare. By using such a dataset, we here provide crucial evidence for the negative consequences of masting breakdown for beeches through reduced pollination efficiency and increasing predation risk. Using a new methodology, we further underline the strong effects of weather cues on reproduction, while showing that changing climate alone cannot be driving the masting breakdown and must interact with currently unidentified factors.

## Graphical abstract

Synchrony and inter‐annual variation in annual seed production of European beech ( *Fagus sylvatica* ) strongly declined over time, resulting in a masting breakdown. As a consequence, predation rsik for seeds strongly increased, while pollination success declined. Seed production is driven by different climatic cues, but changes in these cues can only partly explain the observed masting breakdown.

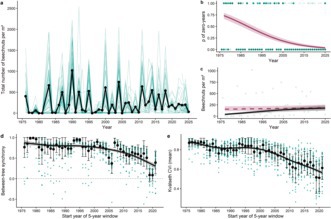

## 1 Introduction

Many perennial plant species reproduce by mast seeding (or masting), i.e., individual seed production is highly variable between years but strongly synchronised with conspecifics within a year, causing total annual seed output to vary between high and low numbers of seeds produced (Herrera et al. 1998; Kelly 1994). Driven by weather cues, masting can be sensitive to climate change (Bogdziewicz et al. 2021) and changes in the magnitude and frequency of seed production can have strong effects on the fitness of the plants themselves (Bogdziewicz, Kelly, Thomas, et al. 2020), as well as on a variety of animal species that depend on the seeds they produce (Dri et al. 2025; Ostfeld and Keesing 2000).

Seeds, such as acorns, chestnuts or beechnuts, provide a substantial food resource for a variety of consumer species with strong bottom‐up effects on their population dynamics (Dri et al. 2025; Perdeck et al. 2000; Touzot et al. 2020; Zwolak et al. 2024). Through the high inter‐annual variability in seed production, masting creates a satiation‐starvation cycle, in which seed consumers get satiated in years of high seed crop, increasing consumer populations, and starved in the following year of low seed production, reducing consumer populations again (Janzen 1971). Thereby, predation pressure is regulated by year‐to‐year variation in seed production, thus enhancing the chance of surviving seeds in high seed years. If seed production changes in magnitude and becomes less variable between years, the satiation‐starvation cycle becomes less pronounced and predation pressure increases. Such fitness benefits, where high reproductive investments yield larger returns, are defined as economies of scale (EOS) and ultimately reduce the costs per surviving offspring (Kelly and Sork 2002; Norton and Kelly 1988; Pearse et al. 2016).

Another well‐supported EOS, besides predator satiation, is pollination efficiency: through the synchrony of tree reproduction, and hence the simultaneous flowering, the amount (Kelly 1994; Moreira et al. 2014) and genetic diversity (Ascoli et al. 2021) of pollen in the air are maximised, which increases the chances of successful pollination in the wind‐pollinated species. Following the pollination efficiency hypothesis (Kelly 1994), a reduction in synchrony is expected to reduce successful pollination and thereby seed production. Pollination can further be affected by climate change, as it is sensitive to weather events, such as heavy rainfall (Tradowsky et al. 2023), that can reduce pollination success (Nussbaumer et al. 2020). These EOS make masting an adaptive reproductive strategy, despite the selective disadvantages of missed reproductive opportunities in years where no seeds are produced, and increased density‐dependent competition in years of high seed production (Bogdziewicz et al. 2024). The effectiveness of EOS is, therefore, crucial for masting to remain beneficial, leading to selection for high inter‐annual variability and high reproductive synchrony among individual trees (Bogdziewicz, Kelly, Tanentzap, et al. 2020; Pesendorfer et al. 2024). With changes in climate, these masting dynamics can change (Foest et al. 2024), but the consequences of these changes for the effectiveness of economies of scale remain understudied.

Masting trees respond to environmental cues (i.e., environmental signals in certain periods of the year) to initiate reproduction and individuals must respond similarly to the same cue to reproduce synchronously (Pearse et al. 2016). Identifying such cues is crucial for understanding the proximate drivers of masting and forecasting how masting may change with changing climate conditions (Journé et al. 2023). So far, many studies (Bogdziewicz, Kelly, Thomas, et al. 2020; Nussbaumer et al. 2020; Övergaard et al. 2007) have tested for climatic effects on seed production by using climate variables from periods that are mostly arbitrarily predefined as selected calendar months (e.g., summer being June and July). Only recently, studies have tested for the exact periods in which trees are most sensitive to climatic cues (Bogdziewicz et al. 2021; Bogdziewicz, Journé, et al. 2023; Journé et al. 2024), resulting in several different methodologies to identify cueing windows (Journé, Simmonds, et al. 2025). While all of them give good approximations of these windows for a single climate variable (Journé, Simmonds, et al. 2025), they mostly do not consider the complex interplay of different climate variables on seed production (but see: Journé, Kelly, et al. 2025). Further development of cue identification methodologies is therefore needed to incorporate the effects of different weather cues.

For European beech ( *Fagus sylvatica* , hereafter beech), most commonly reported weather cues are temperatures and precipitation in the two summers preceding seed fall (Bogdziewicz, Kelly, Tanentzap, et al. 2020; Journé et al. 2024; Vacchiano et al. 2017), with the opening of the temperature window fixed on the summer solstice (Journé et al. 2024; Journé, Simmonds, et al. 2025). Beech is an important forest‐forming species in Central Europe (Leuschner and Ellenberg 2017), which shows high spatiotemporal stability in its cue sensitivity (Bogdziewicz, Journé, et al. 2023), inducing synchrony in reproduction over large distances, since weather conditions are also synchronous over large spatial scales (Koenig 2002). However, trees still vary in their reproductive output despite experiencing the same climate, indicating that weather alone cannot drive masting and suggesting an interplay with internal resource dynamics (Kelly et al. 2025; Müller‐Haubold et al. 2015; Pearse et al. 2016). Depending on resource availability, trees allocate a fraction of the acquired resources to reproduction and reproduction can only be triggered by a cue if the accumulated resources reach a minimum threshold (resource budget model; Crone and Rapp 2014; Schermer et al. 2019). If resource levels are low, reproduction can be largely suppressed even in the presence of strong cues, leading to reduced seed production (Kelly et al. 2025). When cue frequency increases, as through climate change, the interval between cues is not sufficient to replenish depleted resources after reproduction and reproductive output is reduced.

Such climate change effects on masting patterns are already visible in many beech populations where high seed years became more frequent (Sweden: Övergaard et al. 2007, Germany: Gruber 2003; Schmidt 2006, Switzerland, United Kingdom: Nussbaumer et al. 2016) and both inter‐annual variability and synchrony of seed production have declined (Bogdziewicz, Kelly, Tanentzap, et al. 2020; Foest et al. 2024, 2025). This so‐called *masting breakdown* can ultimately lead to strong declines in the fitness benefits of masting for the trees because EOS are becoming less effective, leading to less successful pollination and higher predation (Bogdziewicz, Kelly, Thomas, et al. 2020; Bogdziewicz, Kelly, et al. 2023). These negative consequences of changing masting patterns have so far only been shown in a spatially limited set of beech populations of the UK (Bogdziewicz, Kelly, Thomas, et al. 2020; Bogdziewicz et al. 2021), as long‐term, individual‐based data are needed that include information on pollination and predation rates, which are still rare due to the logistical challenges and high‐effort data collection. It is therefore important to assess whether these climate‐induced changes in masting equally reduce the selective benefits of masting across the species range of European beech.

We here use an individual‐level, 50‐year dataset on beechnut production in the Netherlands to investigate: (1) whether synchrony in and inter‐annual variability of seed production changed over time, and if so, how, (2) which consequences this had on the selective benefits of masting, specifically in relation to pollination and predation, (3) which periods of temperature and precipitation within the year are most influential for beechnut production, using a novel approach of identifying the windows of highest sensitivity, and (4) whether temporal changes in the weather in these windows can explain the changes in masting patterns we observe.

## 2 Materials and Methods

### 2.1 Study Site and Data Collection

Beechnuts have been collected annually from 1976 to 2025 in the Hoge Veluwe National Park, Netherlands (52.038° N, 5.857° E), in a study area located in a mixed forest with poor sandy soils. The same set of 30 to 35 trees was sampled once each year in mid‐October. In total, 81 individual trees have been sampled throughout the study period; trees that were removed through forest management practices or death were replaced by neighbouring trees of similar size. There was also additional sampling effort in a few years (Supporting Information A; Figure S1). The sampled trees were of unknown age, but the diameter at breast height (DBH) of the 35 trees sampled in 2025 (range: 29–148 cm; mean = 65.68 cm) suggests that the smallest trees were at least 70 years old, considering the poor soil conditions they grow on. Based on forest management records from the Hoge Veluwe National Park (pers. comm.), we further estimate that trees at the start of the data collection were also approximately 70 years old, so that all trees were of sufficient age to show masting (Pesendorfer et al. 2020).

Beechnuts were sampled by placing four metal squares (0.25 × 0.25 cm; 0.0625 m^2^) underneath each tree in a straight line of a fixed direction. The first square is placed half a meter from the trunk, the last square below the tip of the largest overhanging branch and the remaining two plots at equal distances in between. In each plot, all full and partial nuts within a square are collected and categorised into either full, empty or predated (showing signs of caterpillar damage, pecking by birds, or gnawing by rodents), and the number of nuts per category is counted. For a more detailed description of the study site and data collection see Jantzen and Visser (2026). This method cannot exclude a potential bias through predation, e.g., by ungulates, or dispersal of nuts by caching species before collection, leading to an underestimation of the absolute number of nuts predated. However, generally the crop size estimates of the ground plot method can be considered highly reliable (Chianucci et al. 2021; Tattoni et al. 2021; Touzot et al. 2018), especially when sampling is done close to peak seed fall, as we do. For the analyses, nuts in each category of all four plots per tree per year are summed and multiplied by four to get estimates of the number of nuts per square metre. Permission for field work was granted by the Hoge Veluwe National Park.

### 2.2 Climate Data

Data on daily minimum and maximum temperatures and daily precipitation sums were retrieved from the Royal Dutch Meteorological Institute (KNMI) for the weather station De Bilt as the closest weather station to the study area (~47 km) for which homogenised data are available (i.e., corrected for changes in measurement methods and relocation of the weather station; Note: the correlation of the mean daily temperatures between De Bilt and Deelen, a weather station within 1 km of the study area, is *r* = 0.994.) Mean daily temperature was calculated as (minTemp + maxTemp)/2.

### 2.3 Statistical Analyses

All statistical analyses were done in R (version 4.5.2) (R Core Team 2025). Generalised linear mixed models (GLMMs) were fitted with *glmmTMB* (version 1.1.13, Brooks et al. 2017) and generalised additive models (GAMs) were fitted with *mgcv* (Wood 2011). Model fits were assessed with the packages *DHARMa* for GLMMs (Hartig 2024) or *gratia* for GAMs (Simpson 2022). Model predictions for GLMMs were calculated with *ggeffects* (Lüdecke 2018).

#### 2.3.1 Masting Metrics

We tested whether the annual seed production of individual trees has changed over the study period by modelling annual total beechnut counts (sum of full, empty and predated nuts) per square meter per tree as the response variable, rather than using decadal averages (as suggested by Bogdziewicz, Kelly, et al. 2023). Although the large between‐tree and between‐year variation in seed production included in annual values might mask part of the temporal trend in seed production, we were also interested in temporal changes in the occurrence of years without reproduction, which the decadal averages did not capture. We therefore use a zero‐inflated negative binomial GLMM to model both the probability of a year without reproduction (zero‐inflation model; binomial) and the trend in the number of nuts produced, if there is reproduction (conditional model; negative binomial). When these two models are taken together, the temporal trend in annual seed production can be estimated. Year as a continuous variable was fitted both as fixed effect and as a zero‐inflation parameter. We fitted tree identity (tree ID) as a random intercept to account for multiple measurements for a given tree over time and accounted for the effect of previous year's seed production (Pesendorfer et al. 2020) by fitting the number of nuts produced by the considered tree of the previous year (i.e., lag‐1 nuts) as a fixed effect.

To assess temporal trends in inter‐annual variation and synchrony in beechnut production, we used a sliding window approach with a window size of 5 years and 1 year step size (similar to Foest et al. 2025). Within each window, synchrony was calculated as the Pearson correlation coefficient in annual beechnut production for each pair of trees. Tree‐level synchrony was then calculated as the mean Pearson coefficient over all pairwise correlations and population‐level synchrony as the mean over all trees (for each window). Interannual variability was calculated as the coefficient of variation (CV_i_) of annual seed production for each tree (i) within each window. Since the commonly used Pearson CV_i_ (^P^CV_i_; =SD/mean) has been criticised as being sensitive to outliers (Lobry et al. 2023), we additionally calculated the newly proposed Kvålseth's CV_i_ (^K^CV_i_; =sqrt (^P^CV_i_^2^ / (1 + ^P^CV_i_^2^)); Kvålseth 2017), which is a stabilised transformation of Pearson CV bounded between 0 and 1. For comparability with other studies, we report ^P^CV_i_ in Supporting Information B. Because we do not expect synchrony and ^K^CV_i_ to change linearly over time, temporal trends were modelled using generalised additive models (GAM) with restricted maximum likelihood (REML) and first year of the sliding window as a smoothing term. For this part of the analysis, trees were excluded from a window if they had less than three out of 5 years of observations in that window.

#### 2.3.2 Economies of Scale

To test for the selective benefits of masting for the trees through economies of scale, we used binomial GLMMs with a logit link function, first‐order temporal autocorrelation with a 1‐year time lag to account for an effect of the previous year's pollination and predation ratio, respectively, and tree ID as a random effect. We combined the hypotheses of the *satiation effect*, which predicts that a smaller proportion of nuts is eaten when the number of nuts is high, and the *starvation effect*, which predicts that a smaller proportion of nuts is eaten in a year when the previous year had much fewer nuts, in one model. In that model, the number of nuts predated against the total number of nuts was fitted as the response variable (using the *cbind*() specification in R), and explanatory variables were the total number of nuts, year, their interaction, their quadratic terms, the ln‐transformed ratio of total number of nuts in year T to the total number of nuts in T1 (i.e., ln (ratio + 1) to avoid logarithms of zero), its interaction with year and its quadratic term. Predated nuts included all nuts predated by caterpillars and other animals (e.g., rodents and birds).

To test the *pollination efficiency* hypothesis, which predicts higher pollination success under high flowering synchrony between individuals and high flower production of conspecifics, full nuts (including full and predated nuts) were used as a proxy for successful pollination, because beechnuts can only develop a kernel when pollinated (Nilsson and Wastljung 1987). Hence, the response variable (proportion of successfully pollinated nuts) was defined as the total number of full nuts against the total number of nuts (using *cbind*() specification). Fixed effects were within‐site within‐year synchrony (defined as the coefficient of variation within year between trees; CVp), year, their interaction, and the quadratic term of year.

For both models, the non‐significant quadratic term for year was dropped, because this was only fitted to explore whether there was a non‐linear temporal trend. The other quadratic terms in the predation model were kept, even when not significant, as they were fitted based on a priori assumptions of non‐linearity. Year as an explanatory variable was used as an ordinal variable to avoid convergence problems and data was restricted to observations with at least one nut (as otherwise proportions cannot be calculated), resulting in *n* = 1048 observations (i.e., tree‐year measurements) for the pollination model, while for the predation model, infinite and non‐defined values for the log (nut ratio) were additionally filtered out (*n* = 691 observations).

#### 2.3.3 Sensitivity Windows for Climate Variables

To model the effects of climate variables on the annual beechnut production, we first identified the period of the year in which seed production is most sensitive to the respective climate variables. First, to create a base model, climate variables over 3 years (T0 = year of seed fall, T1 and T2 = one and 2 years before seed fall, respectively) were calculated for windows commonly described in the literature (e.g., Bogdziewicz, Kelly, Thomas, et al. (2020)): maximum temperature and precipitation sum in summer (June & July) of T1, associated with differentiation of flower primordia (Gruber 2003), and in summer of T2, associated with resource accumulation (Richardson et al. 2005), as well as mean temperatures in the growing season (1 May to 31 August) of T0, and precipitation sum in spring of T0 (1 March to 30 April), associated with flower abortion and pollination success (Nussbaumer et al. 2020). These climate variables were fitted as fixed effects in a zero‐inflated negative binomial GLMM (as used in section (a) for temporal trends in seed production) with annual total beechnut count per square meter per tree as the response variable, previous year's seed production as a fixed effect, tree ID as a random effect, the nbinom2() function with a log‐link was used to specify the negative binomial error distribution, and the zero‐inflation formula included all explanatory variables (=base model). Note that this approach takes into account the error structure of the data (zero‐inflated negative binominal) whereas commonly used approaches do not (see also below). This base model was used as the start model for an iterative sliding‐window approach, in which we tested all possible windows between spring equinox (21 March) and fall equinox (22 September) of a window length between one and 20 weeks (i.e., 7 to 140 days, 15,343 tested windows). For each window, the mean of the minimum, maximum and mean daily temperature, and the sum of the daily precipitation sum were calculated and z‐transformed (i.e., X—mean/SD within window over the years of the study) to bring the variables on the same scale and minimise convergence problems.

To find the best window for the first climate variable (temperature in T1), we fitted a separate GLMM for every window (=15,343 models) by modifying the base model so that temperature in T1 varied according to the window tested. We tested all models separately for minimum, maximum and mean temperature in T1 to determine which temperature measure describes seed production best. For that, we chose the best window for each of them based on the lowest AIC value (Burnham and Anderson 2002), respectively, compared the AIC values of the three best models, and chose the climate variable with the lowest AIC to be used in all further models (this test was only done in the first iteration). For the next focal climate variable, we then replaced the values for temperature in T1 in the base model with the ones of the selected best window. The same procedure was used for temperatures in T2 and T0 (again also testing whether mean, minimum or maximum temperature is best suited) and next, precipitation in the 3 years. After all climate variables of the base model have been exchanged for the values in their respective best windows of the first iteration, we performed a second, third and fourth iteration in the same way. In the starting models of each iteration, all non‐focal variables were set to the values of their respective best windows of the previous iteration. This iterative approach ensured that the initial order in which variables were tested did not influence the final windows selection. Since the best windows of the fourth iteration clearly indicated a best window and only showed slight deviations from the ones of the third iteration, if any, we did not run further iterations (details on the models are shown in Table S1).

Sliding windows have been shown to be a robust and reliable way to identify sensitivity windows for masting (Journé, Simmonds, et al. 2025). The models used within the sliding window approach differ, however, between studies and mostly either calculate correlation coefficients (Journé et al. 2024) or linear regressions (Journé, Simmonds, et al. 2025) between one climate variable and the number of beechnuts per window. Since these approaches do not account for the interplay between different climatic drivers and the highly zero‐inflated nature of masting data, we used a more complex model to address these limitations. For comparability with previous studies, we additionally applied the approach used in Journé et al. (2024) to our data (see Supporting Information C.3 and Figure S4 for more details on comparing both approaches).

#### 2.3.4 Climatic Effects on Seed Production

Since collinearity between all six climate variables was low (absolute *ρ* < 0.4; Figure S5, Supporting Information C.4), we fitted the six climate variables tested above, all set to their respective best window, back into the GLMM used for the sensitivity window analysis to test how each affects annual seed production (=final model). We corrected *p*‐values of each climate variable for the number of windows (*n* = 15,343) tested using a Bonferroni correction. Model fit was assessed by comparing observed and predicted values using a Spearman rank correlation and visually comparing seed production patterns. To better understand whether there is unexplained temporal variation, we additionally fitted year back into the model. Temporal trends in climatic variables were modelled with linear models of each climate variable against year.

## 3 Results

### 3.1 Masting Metrics

**FIGURE 1:**
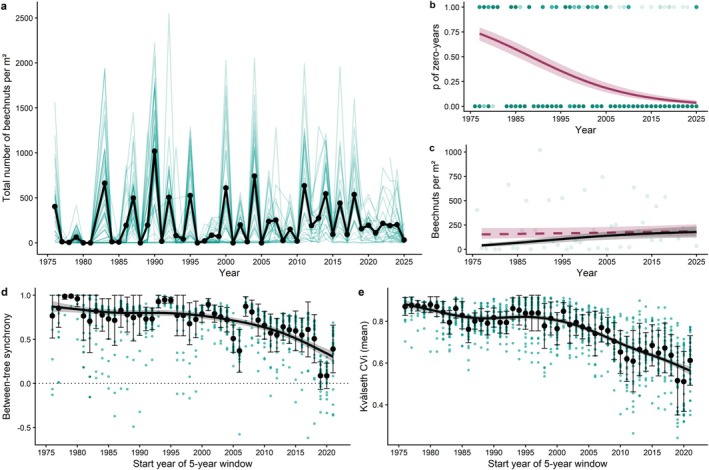
Temporal changes of masting patterns. (a) Total annual number of beechnuts per individual tree (*n* = 81; coloured lines) and the population mean (black). In 1982, no data was collected. (b, c) Model predictions of the temporal trend of annual beechnut production of the population are based on a zero‐inflated negative binomial GLMM: (b) The predicted probability of the population to not reproduce at all in a year decreases significantly over time, indicating that zero‐years become less likely (based on zero‐inflation part of model, red line). Dots show raw data per tree, with 1 being a year without nuts (probability of 1 to be zero) and 0 a year with at least one nut (probability of 0 to be zero). Dots are transparent and darker dots indicate many overlaying data points. (c) The number of beechnuts produced by the population, if reproduction occurs, does not change significantly over the study period (red, dashed line, based on conditional part of model). However, when considering that years without beechnuts are getting rarer (see b), the annual seed output does show a positive trend over time (black, solid line). (d, e) A sliding window approach with five‐year window size and a step‐size of 1 year was used to assess the temporal trend in (d) between‐tree synchrony (i.e., Spearmann correlations between conspecifics) and (e) inter‐annual variation (i.e., Kvålseth CV_i_) of total beechnut production. Raw data per tree is shown as coloured dots (excluding trees from a window when they have less than three observations), population mean as black dots together with their standard deviation. Fitted lines in (d) and (e) are based on model predictions of GAMs with the population mean as a response.

Over the study period of 50 years, seed production of 81 trees has been recorded. Annual seed production alternated between high seed years and years without any reproduction for at least part of the study period (Figure 1a). The probability of a year in which no nuts were found (i.e., a zero year) decreased over time (*β* = −0.089 ± 0.007, *z* = −13.49, *p* < 0.001; Figure 1b), whereas there was no clear change in the number of seeds produced per tree, for years in which a tree produced at least one nut (*β* = 0.004 ± 0.004, *z* = 1.12, *p* = 0.264; Figure 1c, red line). When integrating these effects, i.e., the probability of seed production and the average number of seeds produced per tree when there are seeds produced, seed production of the population shows a slightly increasing trend (Figure 1c, black line). The previous year's seed production (T1) negatively influenced seed production in the current year (T0), when nuts where produced (*β* = −0.0009 ± 0.0001, *z* = −7.268, *p* < 0.001), and increased the probability of a year without nuts in T0 (*β* = 0.001 ± 0.0002, *z* = 5.654, *p* < 0.001).

For the analyses of synchrony and inter‐annual variability (CV_i_), 46 windows of five years were used in the sliding window approach, each containing at least 24 trees. The between‐tree synchrony of beechnut production in the population changed significantly throughout the study period (effective degrees of freedom (edf) = 3.325, *F* = 16.7, *p* < 0.001, *n* = 46), decreasing from between 0.77 and 0.98 in the early windows to around 0.39 in the last window (starting 2021; Figure 1d). Already in the two windows starting in 2005 and 2006, respectively, synchrony showed a sudden strong decline but increased back to its previous level afterwards until there is a second, even stronger decrease in synchrony in the windows starting in 2019 and 2020, where trees have fallen out of synchrony almost completely with a mean synchrony of 0.09. Beechnut production of the population additionally became less variable between years (edf = 4.517, *F* = 53.89, *p* < 0.001, *n* = 46), with the mean ^K^CV_i_ decreasing from around 0.87 at the start of the study period to around 0.61 in the last window (Figure 1e). Similar to synchrony, the windows starting in 2019 and 2020 show a stronger drop in ^K^CV_i_ to a value of 0.51. For ^P^CV_i_ we found similar results, see Supporting Information B (Figure S2).

### 3.2 Economies of Scale

**FIGURE 2:**
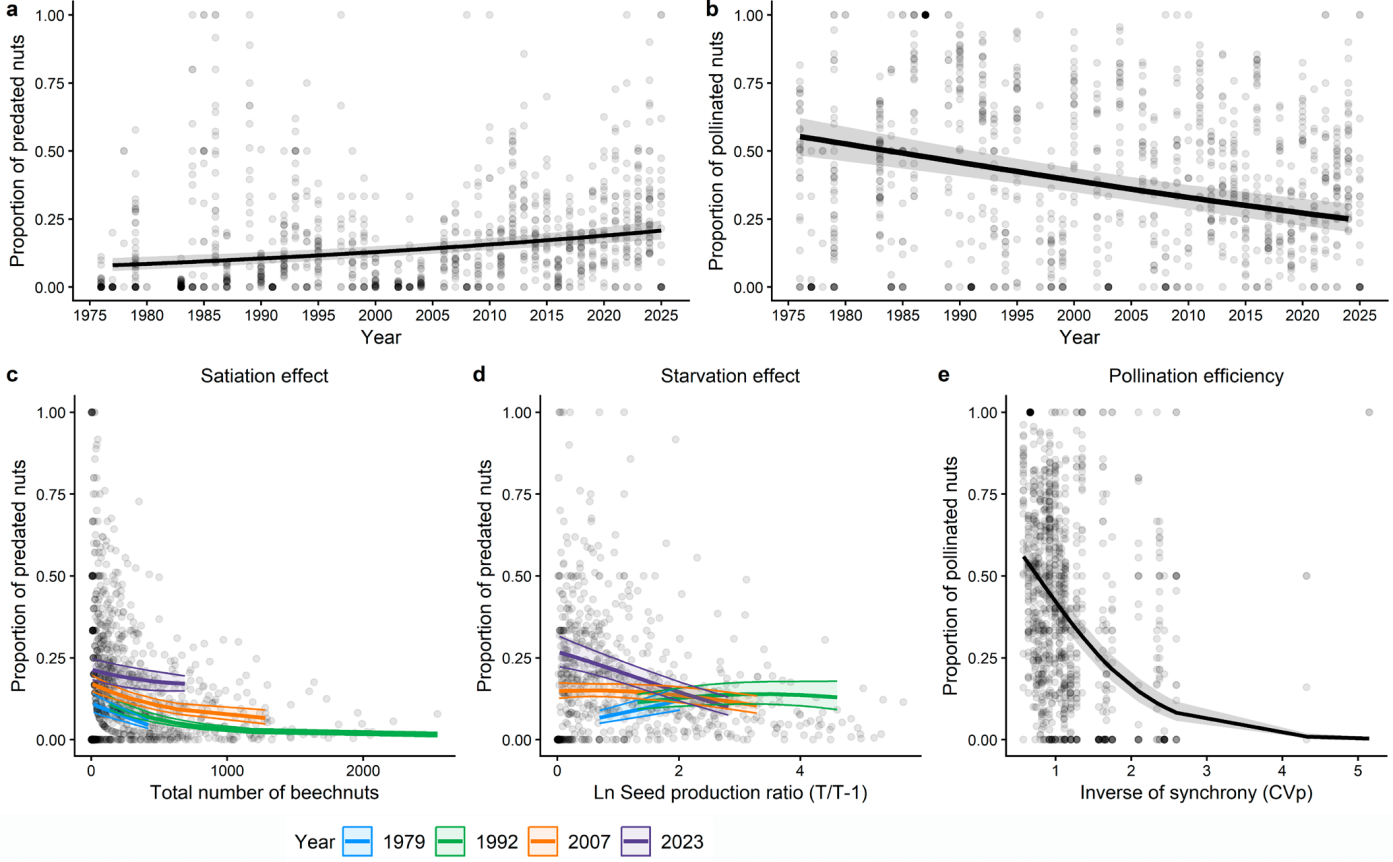
Economies of scale. (a) The proportion of predated nuts strongly increased over time, while (b) the proportion of pollinated nuts declined. The proportion of predated seeds per tree changes with the (c) total number of nuts produced by that tree and (d) the ratio of seeds produced in 1 year to the seeds produced the previous year. For both relationships, these effects changed over time (coloured lines show model predictions for four different years and are truncated to the range of observed values of the respective year). The proportion of effectively pollinated nuts declines the less synchronous trees reproduce, which is shown in (e) with the coefficient of variation (CVp) in reproduction within year between trees (i.e., the inverse of synchrony, meaning that a low CVp indicates high synchrony and vice versa). All lines are fitted based on model predictions of binomial GLMMs.

The proportion of predated nuts increased from 6% in the beginning of the study period to 20% in 2025 (*β* = 0.04 ± 0.006, *z* = 7.167, *p* < 0.001; Figure 2a). In line with the predictions of the *satiation effect*, the proportion of predated nuts declined with the total number of nuts produced and this relationship was better described by a linear than a quadratic term (linear term: *β* = −0.0027 ± 0.0007, *z* = −3.755, *p* < 0.001, quadratic term: *β* = 4 × 10^−7^ ± 3 × 10^−7^, *z* = 1.498, *p* = 0.134) and changed over time (interaction: *β* = 0.00004 ± 0.00001, *z* = 2.928, *p* = 0.003; Figure 2c). Additionally, there is evidence for a *starvation effect*, since the proportion of predated nuts changed with the ratio of seed production in two consecutive years (quadratic term: *β* = −0.056 ± 0.028, *z* = −1.990, *p* = 0.047, linear term: *β* = 0.727 ± 0.204, *z* = 3.566, *p* < 0.001; Figure 2d). In the beginning of the study period, the proportion of predated nuts increased when there were few seeds in the previous year and many seeds in the current year, whereas this relationship was reversed in the second half of the study period (interaction: *β* = −0.021 ± 0.004, *z* = −4.866, *p* < 0.001).

*Pollination efficiency*, measured as percentage full nuts, strongly decreased over time (*β* = −0.027 ± 0.004, *z* = −6.114, *p* < 0.001) from around 55% in 1976 to 25% in 2025 (Figure 2b). The proportion of successfully pollinated seeds decreased as trees became less synchronous (here modelled as the inverse of synchrony, i.e., CVp: *β* = −1.308 ± 0.113, *z* = −11.566, *p* < 0.001; Figure 2e).

### 3.3 Sensitivity Windows for Climate Variables

While the best windows for individual climate variables still shifted strongly between the first and second iteration, they started stabilising in the third iteration and showed almost no change from the third to the fourth iteration (maximum shift was 3 days; see Table S2 for details on all iterations). Maximum daily temperature was identified as the best temperature measure for T1 and T2 (ΔAIC = 40.73 to mean temperature and ΔAIC = 5.38 to minimum temperature for T1, ΔAIC = 33.95 and ΔAIC = 15.06, respectively, for T2) and mean daily temperature for T0 (ΔAIC = 19.49 to maximum temperature, ΔAIC = 51.26 to minimum temperature). For all climate variables in the fourth iteration, there was one distinct trough in the AIC values when plotted against start day of each of the 15,343 windows tested, making us confident that the selected windows (i.e., windows with the minimum AIC) are not random (Figure S3).

**FIGURE 3:**
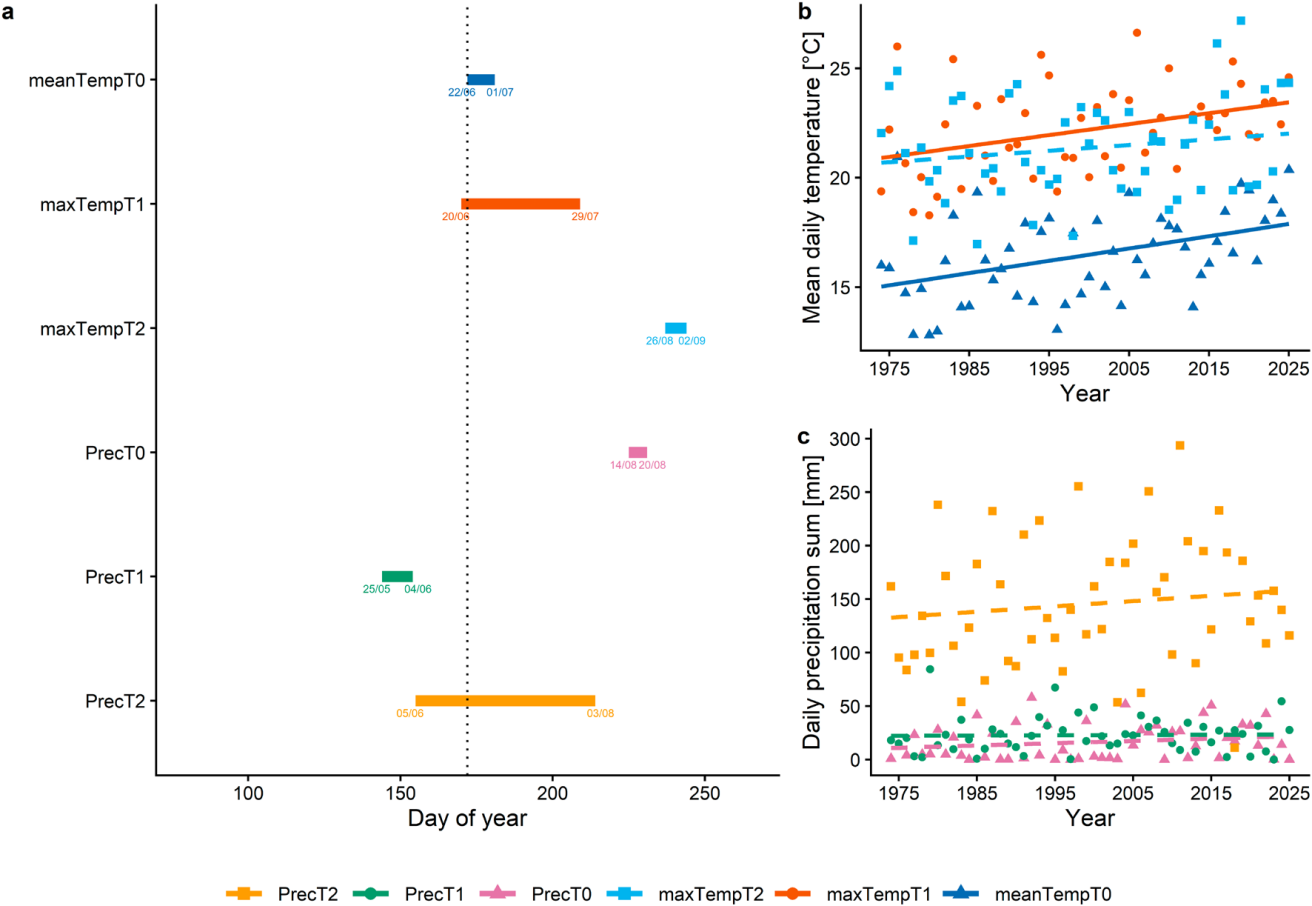
Sensitivity windows and temporal change of climate variables in these windows. (a) Beechnut production is sensitive to temperature and precipitation from different periods in the year of seeding (T0), one (T1) and two (T2) years prior. These sensitivity periods differ in length and are spread across the summer months. Point labels indicate the calendar date, assuming it is not a leap year (as dd/mm) and the dotted line indicates the summer solstice (DOY 172). (b) While mean daily mean (for T0) and maximum (for T1) temperatures in these windows significantly increased over the study period, there is no significant change in maximum temperatures of T2 and (c) the sum of daily precipitation in the sensitivity periods for the 3 years.

Window size was specific to each climate variable, with a minimum of 7 days (for precipitation in T0) and a maximum of 60 days (for precipitation in T2). In the year of seeding, beeches showed the highest sensitivity to mean temperatures in late June (22/06 to 01/07) and precipitation in the second half of August (14/08 to 20/08). In the year prior to seeding (T1), maximum daily temperatures of late June to the end of July (20/06 to 29/07) and precipitation in late May (25/05 to 04/06) were most relevant, while 2 years prior to seeding (T2), late summer temperatures (26/08 to 02/09) and summer precipitation (05/06 to 03/08) were most related to seed production (Figure 3a).

### 3.4 Climatic Effects on Seed Production

Throughout the study period, only temperatures in the best windows of T0 and T1 have significantly changed (T0: *β* = 0.028 ± 0.008, *t* = 3.273, *p* = 0.002; T1: *β* = 0.025 ± 0.009, *t* = 2.868, *p* = 0.006; Figure 3b), while there was no significant change in temperature in the window for T2 (*β* = 0.011 ± 0.009, *t* = 1.224, *p* = 0.227) and precipitation sums in any of the best windows (T0: *β* = 0.008 ± 0.009, *t* = 0.903, *p* = 0.371; T1: *β* = 0.013 ± 0.009, *t* = 1.414, *p* = 0.163; T2: *β* = 0.001 ± 0.009, *t* = 0.139, *p* = 0.890; Figure 3c).

Fitting all these climate variables set to their respective best windows of the fourth iteration into the final model showed that a cold and wet climate in T2, a warm and dry climate in T1, and a warm and wet climate in T0 (despite the temperature effect not being significant) increase the number of nuts produced, if a tree reproduces (conditional model, Table 1). The probability of a tree not reproducing in a year was also significantly affected by all climate variables, with cold temperatures in the periods of T0 and T1 and warmer temperatures in T2 increasing the probability of a year without reproduction, as well as dry periods in the windows of T0 and T2, and wet periods in T1 (zero‐inflation model, Table 1; note that positive estimates indicate that an increase in the fixed effect leads to a higher probability of a zero‐year, i.e., no reproduction, and vice versa for negative estimates). Fitting year back into the model showed that there was no unexplained variation remaining for the conditional model (*β* = 0.0009 ± 0.0031, *z* = 0.276, *p* = 0.783), but year was significant in the zero‐inflation model (*β* = −0.044 ± 0.013, *z* = −3.437, *p* = < 0.001), indicating that the climate variables considered here cannot fully explain the variation in the probability of a year without reproduction.

**TABLE 1:** Drivers of annual beechnut production.

Fixed effects	Conditional model			Zero‐inflation model		
	Estimate (±SE)	*z*	*p*	Estimate (±SE)	*z*	*p*
Mean temperature T0	0.198 (0.043)	4.57	0.076*	−0.800 (0.102)	−7.812	< 0.001*
Max temperature T1	0.306 (0.041)	7.41	< 0.001*	−0.847 (0.126)	−6.713	< 0.001*
Max temperature T2	−0.411 (0.037)	−11.17	< 0.001*	1.494 (0.245)	6.104	< 0.001*
Precipitation T0	0.449 (0.035)	12.95	< 0.001*	−1.993 (0.256)	−7.774	< 0.001*
Precipitation T1	−0.765 (0.044)	−17.49	< 0.001*	2.295 (0.245)	9.380	< 0.001*
Precipitation T2	0.528 (0.039)	13.46	< 0.001*	−2.881 (0.369)	−7.799	< 0.001*
Number nuts T1	−0.0005 (0.0001)	−4.42	< 0.001	0.0008 (0.0003)	2.717	0.007

*Note:* Results from the final, negative binomial zero‐inflated GLMM in which all fixed effects are set to their respective best window. The model included annual beechnut production per tree as the response variable, tree ID as random effect and previous year's number of nuts as a fixed effect. T0 marks the year of seed fall, T1 and T2 1 and 2 years prior, respectively. All fixed effects were z‐transformed within their respective window across the study period and estimates are on the log‐link scale. The zero‐inflation part of the model estimates the probability of a year without reproduction (true zero), with positive estimates indicating that an increase in the fixed effect leads to a higher probability of zero nuts, i.e., no reproduction. The conditional model explains the effects on the number of nuts produced, if reproduction occurs, with positive estimates indicating that an increase in the fixed effect increases the number of nuts produced. *p*‐values for climate variables are corrected by multiplying them with the number of windows tested (p* 15343 windows, corrected *p*‐values are indicated with *).

**FIGURE 4:**
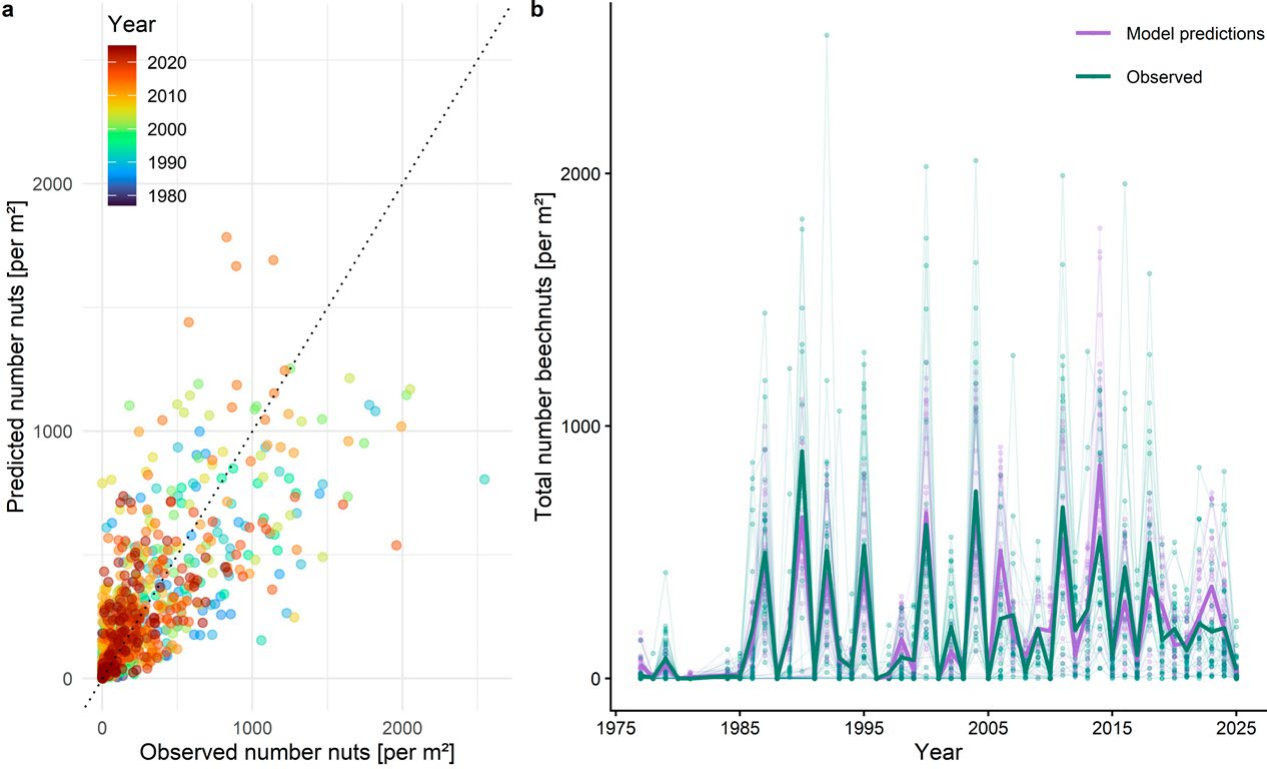
Model predictions and observed number of beechnuts. (a) Comparing the model predictions of the final model with the observed number of beechnuts shows that the model underestimates large seed crops, while it overestimates beechnut production more often in the most recent years. Predicted and observed values are correlated with *ρ* = 0.86. Dots are individual trees; the dotted line represents a perfect correlation. (b) When comparing temporal patterns in observed and predicted beechnut counts, it shows that inter‐individual differences (thin, transparent lines) are less well captured by the model and that temporal patterns, especially in the population mean (thick lines), start diverging more strongly in the second half of the study.

Overall, the final model predicted annual seed production well (*ρ* = 0.86, Figure 4a) but generally underestimated large seed crops. However, consistent with a transition in masting dynamics in the past two decades, the model tended to overestimate the number of nuts produced in recent years and predictions deviated more strongly from observed values. Comparing temporal patterns in observed and predicted seed production (Figure 4b) showed that these were well aligned until the early 2000s, whereas deviations in the later 2000s were caused by a more consistent pattern of peaks and valleys predicted by the model, while peaks declined more strongly in the observed seed production. Although the model captured changes in masting, since peaks in annual seed production became shorter and less frequent over time and predicted seed output rarely reaches zero in the past decades, these predicted changes were less severe than observed changes, and the model, therefore, could not accurately capture the masting breakdown.

## 4 Discussion

Based on 50 years of annual seed production data, we demonstrated that beech trees in the Netherlands show a clear *masting breakdown* beginning around 2008. Since then, years without seed production become highly unlikely, annual beechnut production stabilises at a low number, and between‐tree synchrony and inter‐annual variability in seed production declined strongly. This ultimately led to a strong decline of pollination efficiency and a strong increase in the proportion of predated seeds, since economies of scale became less efficient. In line with the literature (Journé et al. 2024; Kelly et al. 2013; Vacchiano et al. 2017), seed production was driven by temperatures and precipitation across 3 years (year of seed fall and both years before that), but we identified partly different cueing periods for each of these weather variables than previously reported. From the six weather cues, only temperatures in T0 (year of seed fall) and T1 (year prior to seed fall) showed significant warming over time. However, these changes in temperature alone cannot explain the observed masting breakdown, because the final model of annual beechnut production explained by climate cues predicted less extreme changes in seed production.

Reproduction through masting can only be beneficial if costs per surviving offspring are reduced (Kelly and Sork 2002), which is no longer the case when masting breaks down. With smaller seed crops in recent years, satiation of seed consumers became less efficient and resulted in an increase in the proportion of predated beechnuts. Consistent, low‐level seed production should offer a more stable food supply for consumers, which can stabilise or increase seed consumer populations and, thereby, further increase predation pressure. Due to the decline in inter‐annual variation in seed production we would have expected the predator starvation benefits (Janzen 1971) to have weakened in recent years, since the ratio of seed production of two consecutive years became much smaller. In contrast, the starvation effect became seemingly stronger, since the proportion of predated beechnuts is reduced more strongly in recent years if there are many more nuts in one year compared to the preceding year (i.e., T/T‐1 is large). Comparing this relationship between early and late years of the study is, however, difficult. While early years showed a more clustered distribution at the higher end of the range of interannual differences in seed production, the distribution in recent years has become more uniform. Hence, variation in seed production of two consecutive years was never low in early years, but is commonly low in recent years, and overall seed predation is higher now than 50 years ago, making the decline in the predation with increasing seed production ratio more visible now, but the starvation effect not necessarily stronger. Consequently, the changing masting patterns can no longer maintain the mechanisms that reduce the proportion of predated seeds. Here, we only consider pre‐dispersal predation by insects and on‐site predation through birds and rodents, because our seed collection method cannot capture seeds that are removed or eaten whole. While we think that this measure is sufficient to draw conclusions on changes in predation risk and predator starvation and satiation, it can underestimate the effect of predation on the tree's fitness and potentially underestimate seed production in years when (some) predators are more abundant.

Besides stabilising seed production, the breakdown of reproductive synchrony also strongly reduces pollination efficiency. While pollination success is greater than 50% under high reproductive synchrony, it steeply declines the less synchronous trees within the population flower, showing strong support for the pollination efficiency hypothesis (Kelly 1994). Even though masting breakdown is reported throughout Europe (Foest et al. 2024), long‐term individual‐level data on pollination and predation is rare, making our study valuable evidence for the severe consequences of masting breakdown for the selective benefits of masting.

Masting breakdown is driven by changes in the weather cues and their effects on internal resource dynamics (Kelly et al. 2025), which makes identifying these weather cues the first step towards understanding the underlying mechanism of masting breakdown. Summer temperatures in the two years before seed fall have repeatedly been shown to affect beechnut production (Bogdziewicz, Kelly, Thomas, et al. 2020; Kelly et al. 2013; Nussbaumer et al. 2020; Vacchiano et al. 2017), but only recently have cueing windows in summer been more precisely defined to start around the summer solstice (Journé et al. 2024). While we find the same for temperatures in T1, the cueing window for temperature in T2 (2 years prior to seed fall) is much later in summer and, spanning only 8 days, rather short in duration. For T0, we expected spring temperatures to affect seed production (Nussbaumer et al. 2020), but found seed production to be most sensitive to a window that also opened at the solstice. Temperatures in this period did not affect the number of nuts produced, but only whether or not reproduction occurs. Given that seed fall starts in early autumn and seeds are therefore almost fully formed in late summer, the mechanism behind this temperature effect on the occurrence of reproduction remains unclear, as well as the effect of the short and late window for precipitation in T0.

Unlike temperature, precipitation cues were not anchored to the summer solstice, neither in our study nor in previous ones (Journé et al. 2024) and while the window in T1 is unexpectedly short, both precipitation cues of T1 and T2 fall into the expected summer period. All three precipitation cues significantly affected beechnut production in our population, despite precipitation often being deemed lower priority as a driver of seed production than temperature (Drobyshev et al. 2010; Vacchiano et al. 2017). Given that precipitation affects trees through the soil water content, which also depends on local factors (e.g., soil porosity, drainage, water holding capacity, evaporation and vegetation cover; Grayson et al. 1997), precipitation effects are expected to show stronger local differences than temperature. Soil water content would therefore be a better measure of water availability, but such information was not available for this study. Integrating this could strengthen the model of environmental drivers of seed production further, especially because soil water content strongly affects nutrient uptake rates in beeches (Geßler et al. 2005) and thereby also affects resource dynamics. Regarding precipitation, it would further be interesting to investigate whether the strength and prevalence of precipitation anomalies, rather than cumulative or average precipitation values, better explain seed production dynamics and result in the same cue windows.

The differences in weather cues we find here compared to previous studies might reasonably be attributed to methodological differences, rather than biological ones. Several methodologies exist to identify weather cues and have all been shown to reliably detect a benchmark window (Journé, Simmonds, et al. 2025). However, these methods mostly do not consider the interplay of different climatic drivers on seed production, nor do they assess the differential effect of climate on the occurrence of reproduction and the number of nuts produced, if reproduction occurs. Previous studies often log‐transformed seed production data to normalise it (Journé et al. 2024; Journé, Simmonds, et al. 2025), but given the highly zero‐inflated nature of masting data, especially for individual‐level data, this leads to problems associated with log‐transforming count data (O'Hara and Kotze 2010) or exclusion of zeros counts, which are an essential part of masting. Only recently, these limitations were partly tackled by testing several climate variables in beta regression models (using absolute maximum standardisation of seed counts to a 0–1 scale), to identify weather cues (Journé, Kelly, et al. 2025). While this standardisation makes trends between populations more comparable, it does not allow comparison of the magnitudes of effects on masting. Here, we applied an alternative method that accounted for these limitations (i.e., zero‐inflation, several climate variables, comparability of magnitudes) and additionally looked at the different effects of environmental drivers on the number of seeds produced and the occurrence of reproduction. While we found slightly different results using this approach, we were also able to replicate previous findings by combining our data with one of the established approaches (as described in Journé et al. (2024)). As experimental tests on masting are challenging (Bogdziewicz, Ascoli, Hacket‐Pain, et al. 2020), we cannot assess which results are closer to the real weather cues that affect masting, but we argue that the approach we use here is better suited to accommodate the data structure and the complex biological processes driving masting. Testing our approach in comparison to existing approaches across populations would therefore be interesting. Compared to a model using weather cues defined as broad ranges of months (e.g., June–July), as previously done in the literature, which explains only 46% of the variance, the same model using the more precise windows identified by our approach explains 63% of the variance, highlighting the need to identify climatic cues more precisely.

Despite the cueing windows being different, the overall effect of weather on beechnut production is the same as commonly reported: a wet and cold climate in T2, under which resources can build up (Richardson et al. 2005), followed by a dry and warm climate in T1, which enhances the formation of flower primordia (Gruber 2003), and warm and dry weather in T0, when flowers are pollinated, positively affects reproduction. While annual variation in the number of produced seeds, if reproduction occurred, could be fully attributed to temperature and precipitation, these climatic factors alone could not determine whether or not reproduction occurs. Comparing model predictions with observed values of annual seed production showed that the severity of temporal changes in the masting pattern is underestimated by the model, again indicating that there must be other factors, in combination with temperature changes, driving the masting breakdown. One of them could be internal resource dynamics, which can modulate the response to climate cues in beech (Kelly et al. 2025).

Since trees in our population respond to a warm temperature cue in the year before seeding, the increasing temperatures in this cueing window increased cue frequency, which initiates reproduction more frequently (Bogdziewicz et al. 2024). With a shorter period between reproductive events, resources may not sufficiently be replenished (Kelly et al. 2025), weakening the tree's sensitivity to the temperature cue (Bogdziewicz et al. 2021). Trees then produce seeds in proportion to the limited resources that they could build up in this time, leading to smaller, less variable, and more frequent seed output. While trees could benefit from increasing temperatures if they enhance resource availability, allowing them to frequently produce high crops (Kelly et al. 2025), this is not the case in our population, as seed production seems to stabilise around 250 nuts/m^2^. Increased cueing frequency can further magnify inter‐individual differences in resource budgets (Bogdziewicz 2022), inducing different responses to the same cue, ultimately resulting in decreasing synchrony.

Although these interactive effects of weather cues and internal resource reserves in theory could explain the observed changes in masting patterns of our population, we actually do not see a significant effect of resource reserves both on the probability of reproduction and the number of nuts produced when fitting resource reserves into the final model, including all six weather cues (Supporting Information D). Resources neither have a significant effect in interaction with the temperature cue of the year prior to seeding (T1) nor as an additive effect when all weather cues are included, while the interaction with the temperature cue in T1 is significant if all other weather cues, besides temperature in T2, are excluded (following previous work; Kelly et al. 2025). Resources may therefore only weakly modulate the tree's response to weather cues in this population, increasing the tree's sensitivity to climate change effects (Bogdziewicz et al. 2024). The calculation of resource reserves is here based on the cumulative reproductive effort (for more details: Kelly et al. 2025; Rees et al. 2002), but as trees do not only rely on stored resources but also on resource uptake throughout the reproductive cycle (Allen et al. 2017), this measure of resource availability may not sufficiently capture the overall effect of available resources and thereby, mask the relationship with weather cues. Generally, nitrogen fertilisation enhances seed production (Bogdziewicz et al. 2017) and nitrogen levels are highly correlated with the on–off cycle of expression of flowering genes (Miyazaki et al. 2014). Under the predicted increasing anthropogenic nitrogen deposition (Galloway et al. 2004), this suggests a further reduction of the probability of years without reproduction and would thereby strengthen the masting breakdown. A more reliable method of measuring resources (e.g., measuring the nutrient content in reproductive structures (Fernández‐Martínez et al. 2017)), and combining internal and external resource availability into future models describing annual beechnut production is, therefore, crucial to more accurately capture masting breakdown and inter‐individual variation between trees. Consequently, we can currently not conclusively determine which other factors, besides changes in temperature, are driving the observed masting breakdown.

Compared to UK populations, masting breakdown in our population occurred roughly at the same time around the mid‐2000s (Hacket‐Pain et al. 2025), but with 49%, the decrease in synchrony was stronger in our population (compared to 30% in Bogdziewicz, Kelly, Thomas, et al. 2020), while the 30% decline in inter‐annual variability was less extreme (compared to 40% in UK). Despite populations across the species range showing masting breakdowns (Foest et al. 2024), this highlights that the magnitude of these changes can differ between populations, as they can differ in their response to local conditions (as shown in oak in Fleurot et al. 2023). Given the negative fitness consequences caused by the masting breakdown, there is selection for individuals that are highly sensitive to climate cues, because they will show higher synchrony and inter‐annual variability in seed production, and thereby selection for masting (Bogdziewicz, Kelly, Tanentzap, et al. 2020). While this could allow beeches to cope with climate‐induced changes, the long generation time of beech trees and other masting tree species makes it challenging to keep up with the pace of climate change.

Despite looking at several aspects of masting, this first analysis of this valuable long‐term dataset leaves room for more elaborate questions, especially in connection with seed consumer populations. While masting breakdown decreases the fitness of the trees, the more stable seed supply in the forest might positively affect seed consumers, as beechnuts are a crucial food resource for many species, such as rodents (Zwolak et al. 2024), wild boar (Touzot et al. 2020), or great tits (Perdeck et al. 2000). Changes in food supply can have yet unforeseen effects on the forest, as changes in seed consumer population can further cascade through the wider food web (Ostfeld and Keesing 2000). However, given the decreasing pollination rate, many of the produced nuts will be empty and therefore not of use for consumers. Investigating changes in viable seed supply linked to population changes of forest species therefore provides an interesting avenue for further research.

## Author Contributions

**Cherine C. Jantzen:** conceptualization (equal), data curation (equal), formal analysis (equal), methodology (equal), visualization (equal), writing – original draft (equal). **Joseph B. Burant:** conceptualization (equal), methodology (equal), supervision (equal), writing – review and editing (equal). **Marlène Gamelon:** methodology (equal), writing – review and editing (equal). **Elisabeth S. Bakker:** conceptualization (equal), methodology (equal), supervision (equal), writing – review and editing (equal). **Marcel E. Visser:** conceptualization (equal), data curation (equal), methodology (equal), supervision (equal), writing – review and editing (equal).

## Funding

This publication is part of the project *The heartbeat of the forest* with file number OCENW.M.22.426 of the research programme NWO Open Competition Domain Science which is financed by the Dutch Research Council (NWO). M.G. was funded by the French National Research Agency ANR PURE project (ANR‐23‐CE02‐0028).

## Conflicts of Interest

The authors declare no conflicts of interest.

## Supporting information


**Figure S1:** Sampling years per tree. Each row in the raster is representing one of the studied trees and the raster is filled for each year beechnuts have been collected. Data is missing for 1982. While some trees have been sampled over the whole study period, others were only sampled for shorter intervals and often replaced by other trees when they were taken out of the study due to death or cutting. Colours aid the distinction of rows.
**Figure S2:** Pearson CV (^P^CV) of annual seed production. ^P^CV_i_ is calculated using a sliding window approach with a window size of 5 years and 1 year step‐size (see main text). Blue dots indicate the CV_i_ of individual trees, the black dots the population mean per year. There was a significant effect of year based on a GAM fitting ^P^CV_i_ against the year in which the window opened (~s (year)). The fitted line is based on model predictions; the ribbon indicates their standard error.
**Table S1:** Model specifications of iterative process to find sensitivity windows for each climate variable. Each model includes a temperature and precipitation variable for year T0 (year of seed fall), T1 (year prior to seed fall) and T2 (2 years prior to seed fall). Each model is run for every window of a length of 7 to 140 days between March 21 and September 22, resulting in 15,343 windows. The focal variable varies according to the respective window, while the remaining variables are kept constant to a specified window. The window for which each variable is kept constant is specified in brackets: ‘base’ = window commonly defined in the literature (June & July for T1 and T2 and May to August for temperature T0, March to April for precipitation T0), ‘best win In’ = best window found in nth iteration (n can be first, second, third or fourth), and ‘test’ indicating the focal variable that can vary (additionally bold). After model 3, 6 and 9 it was decided based on the lowest AIC value, which of the temperature variables is used (min, max or mean temperature) in further models (i.e., max temperature for T1 and T2, mean temperature for T0). For the second iteration, all variables are set to their respective best window found in the first iteration and each variable is tested again and the same process is used for the third and fourth iteration (i.e., setting all variables to the best window of the previous iteration). All models are zero‐inflated negative binomial GLMMs with tree ID as a random effect, number of nuts of the previous year as fixed effect, and individual‐level total annual number of beechnuts as response variable (see methods c) in main text.
**Table S2:** Best windows from all four iterations. For each climate variable, the start and end days of the best window are given as DOY of the year and calendar date in parentheses (assuming the year was not a leap year), as well as the window length and the AIC of the model for all four iterations.
**Figure S3:** AIC values of all tested windows per climate variable in the fourth iteration. For each of the 15,343 windows tested per climate variable, the AIC value of the respective model is plotted against the start day of the window. For all variables, there is a clear pattern in AIC values with a through around a certain date visible. This indicates that the window with the lowest AIC, which is selected as the best window, is not selected by chance, as all windows around the same date with a similar length have low AIC values as well.
**Figure S4:** Spearman correlation of log‐transformed annual beechnut count and temperatures in a seven‐day sliding window with 1‐day step‐size. The x‐axis shows the day of the year on which the seven‐day window closes, starting at January 1 of year T‐2 (DOY 1) until December 31 of the year of seed fall (DOY 1095). All years are treated as non‐leap years. The dashed lines indicate the day of the summer solstice in all 3 years. Blue dots show positive correlations, red negative correlations. The upper panel shows the correlation with mean daily temperatures (as used in Journé et al. (2024)), the lower panel the correlation with maximum daily temperatures as used in the main analysis.
**Figure S5:** Correlation matrix of the six explanatory climate variables. Numbers show the Pearson correlation coefficient (*ρ*) between each pair of climate variables. Colours facilitate the interpretation of these values, with darker red squares indicating more negative correlations, and darker blue squares more positive correlations. The correlation between climate variables is overall low, allowing to fit all variables into the same model.

## Data Availability

The data and code that support the findings of this study are openly available in Zenodo at http://doi.org/10.5281/zenodo.20542058. Please be aware that the data used here is a derivative of the full dataset on beechnut production in the Netherlands, which can be found on DataverseNL (DOI: http://doi.org/10.34894/TQY74M).
