# Supplementary material for: Masting Breakdown in European Beech Reduces Fitness Benefits of Masting, Partly Explained by Climate Change
Source: Ecol Evol. 2026 Jun 8;16(6):e73809. doi: 10.1002/ece3.73809 (PMC13247300; doi:10.1002/ece3.73809)
Supplement: Supplementary file 1 — Figure S1: Sampling years per tree. Each row in the raster is representing one of the studied trees and the raster is filled for each year beechnuts have been collected. Data is missing for 1982. While some trees have been sampled over the whole study period, others were only sampled for shorter intervals and often replaced by other trees when they were taken out of the study due to death or cutting. Colours aid the distinction of rows. Figure S2: Pearson CV (PCV) of annual seed production. PCVi is calculated using a sliding window approach with a window size of 5 years and 1 year step‐size (see main text). Blue dots indicate the CVi of individual trees, the black dots the population mean per year. There was a significant effect of year based on a GAM fitting PCVi against the year in which the window opened (~s (year)). The fitted line is based on model predictions; the ribbon indicates their standard error. Table S1: Model specifications of iterative process to find sensitivity windows for each climate variable. Each model includes a temperature and precipitation variable for year T0 (year of seed fall), T1 (year prior to seed fall) and T2 (2 years prior to seed fall). Each model is run for every window of a length of 7 to 140 days between March 21 and September 22, resulting in 15,343 windows. The focal variable varies according to the respective window, while the remaining variables are kept constant to a specified window. The window for which each variable is kept constant is specified in brackets: ‘base’ = window commonly defined in the literature (June & July for T1 and T2 and May to August for temperature T0, March to April for precipitation T0), ‘best win In’ = best window found in nth iteration (n can be first, second, third or fourth), and ‘test’ indicating the focal variable that can vary (additionally bold). After model 3, 6 and 9 it was decided based on the lowest AIC value, which of the temperature variables is used (min, max or mean temperature) in fu [file ECE3-16-e73809-s001.docx]

**Supporting information to**

**Masting breakdown in European beech reduces fitness benefits of masting, partly explained by climate change**

Cherine C. Jantzen, Joseph B. Burant, Marlène Gamelon, Elisabeth S. Bakker, Marcel E. Visser

Contents

1. Study site and sampled trees
2. Inter-annual variability: Pearson ^P^CV_i_ and Kvålseth ^K^CV_i_
3. Sensitivity windows

C.1. Limitations of existing methodologies to identify weather cues of masting

C.2. Our approach

C.3. Comparison with approach used in Journé et al., (2024)

C.4. Collinearity test of explanatory climate variables

D. Effect of internal resource reserves on seed production

1. **Study site and sampled trees**

The study site ranges roughly 111 ha and is characterized by a mixed-forest of birch (*Betula*), pine (*Pinus*), oak (*Quercus*), and larch (*Larix*) growing on poor sandy soils (Jantzen & Visser, 2026). Potential seed predators include wild boar (*Sus scrofa*), passerine birds (e.g., great tit (*Parus major*), blue tit (*Cyanistes caeruleus*), chaffinch (*Fringilla coelebs*)), jays (*Garrulus glandarius*), wood pigeons (*Columba palumbus*), red squirrels (*Sciurus vulgaris*) and small rodents (e.g., wood mouse (*Apodemus sylvaticus*) and bank vole (*Myodes glareolus*). Over the 50-year study period, it was aimed to sample the same individual trees each year, but due to forest management practices or death of trees, some had to be replaced by neighbouring trees. Some additional trees have been sampled only in a few years due to different sampling effort, while no data was collected in 1982 (Figure S1).

**
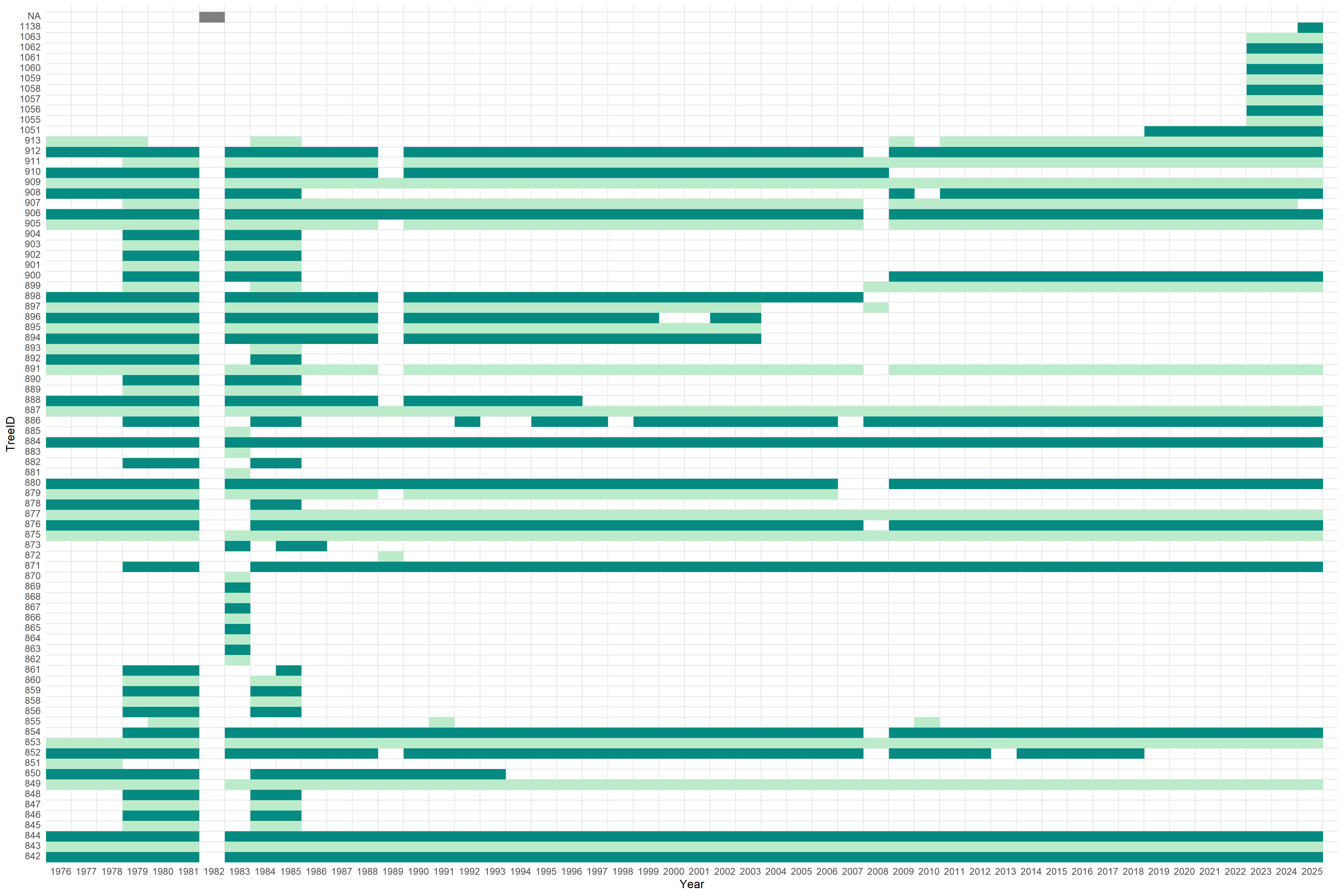
**

***Figure S1 Sampling years per tree.*** *Each row in the raster is representing one of the studied trees and the raster is filled for each year beechnuts have been collected. Data is missing for 1982. While some trees have been sampled over the whole study period, others were only sampled for shorter intervals and often replaced by other trees when they were taken out of the study due to death or cutting. Colours aid the distinction of rows.*

1. **Inter-annual variability: Pearson ^P^CV_i_ and Kvålseth ^K^CV_i_**

The Pearson CV_i_ (^P^CV_i_) shows a significant decrease over the study period (edf = 6.343, F = 41.99, p < 0.001, n = 46). While it was between 1.83 and 1.88 at the beginning of the study period, it declines to 0.82 in the last window starting 2021 (Figure S2). In the two windows starting in 2019 and 2020, there is an even stronger decline to 0.65 and 0.63, respectively. Compared to Kvålseth ^K^CV_i_ reported in the main text, the predicted trend shows more variation, with an initial decline until 1988, followed by a small increase until 1996 and another overall decrease until the end of the study period.


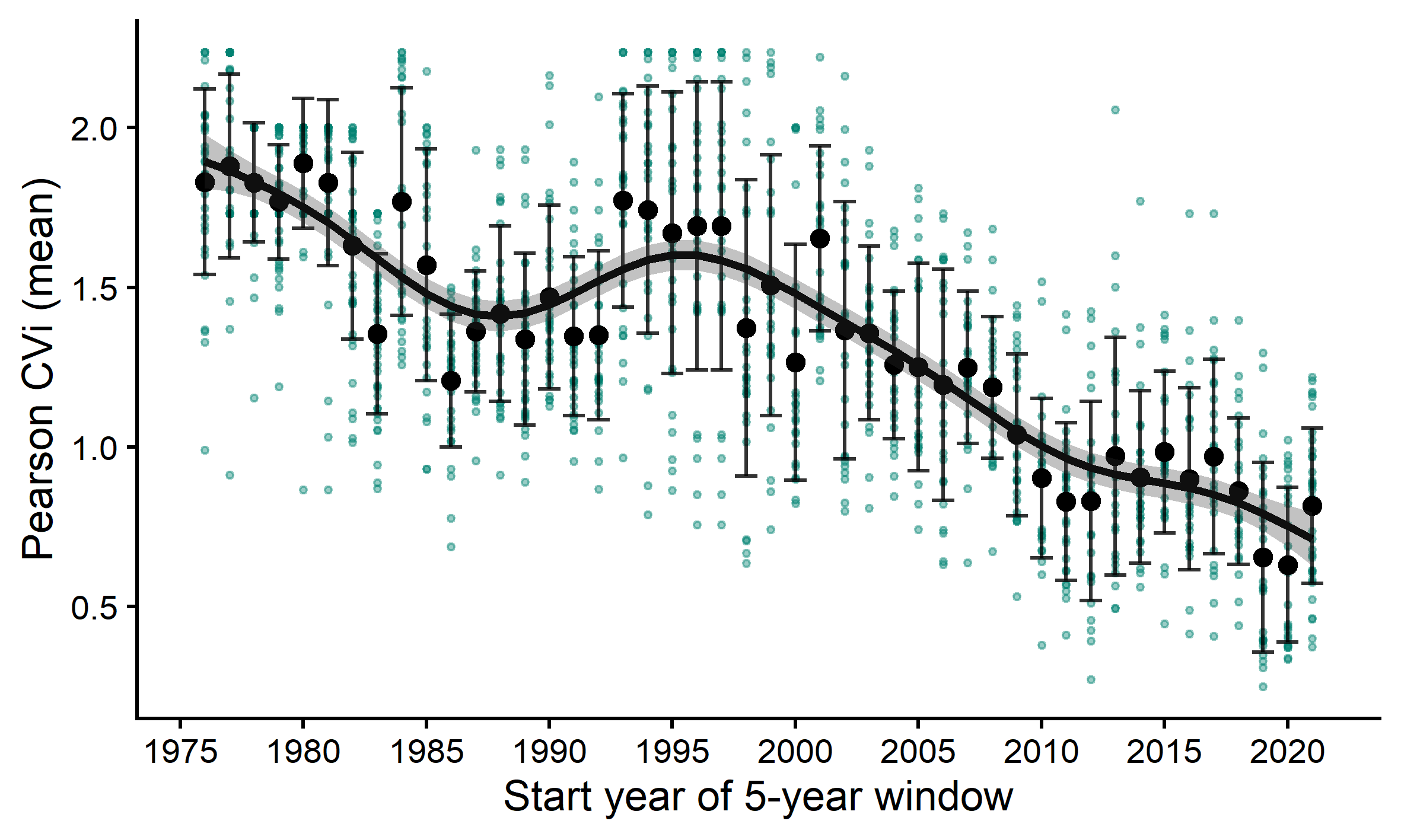


**Figure S2 Pearson CV (^P^CV) of annual seed production.** ^P^CV_i_ is calculated using a sliding window approach with a window size of five years and one year step-size (see main text). Blue dots indicate the CV_i_ of individual trees, the black dots the population mean per year. There was a significant effect of year based on a GAM fitting ^P^CV_i_ against the year in which the window opened (~ s(year)). The fitted line is based on model predictions; the ribbon indicates their standard error.

1. **Sensitivity windows**

**C.1. Limitations of existing methodologies to identify weather cues of masting**

As explained in more detail in the main text, several methodologies exist to identify those periods in the year in which a biological response variable is most sensitive to climate variables (methodologies for finding sensitivity windows of annual seed production are compared in Journé et al., (2025)). Commonly, these methodologies analyse the window of highest sensitivity for each climate variable separately, which neglects the complex interplay of other climatic factors that influence the response variable. Additionally, many methods require normally distributed data, often resulting in normalization of the data by log-transformation or other standardisation methods. For highly zero-inflated data, such as annual seed counts (especially on an individual tree level), log-transformation leads to an exclusion of zero counts (as the logarithm of zero is not defined), which will bias the results. Additionally, most of these methods fit linear regressions or Spearman rank correlations between seed counts and the climate variable, assuming linearity in this relationship, which can only be assumed if zero-counts are excluded. Lastly, climate variables might act differently on the number of annually produced seeds than on whether or not reproduction occurs. This can however be accounted for by using more complex, zero-inflated models, which model these two components separately and integrate their effects.

Most recent approaches start to account for some of these limitations (Journé, Kelly, et al., 2025) by standardising data through absolute maximum standardisation (i.e., dividing each value by the maximum value of the time series), making seed counts bound between 0 and 1, and fitting beta regressions testing multiple climate variables in the same model. This accounts better for the data structure and does not exclude zero-counts, but magnitudes of effects between time series (i.e., populations) become less comparable through this standardisation.

**C.2. Our approach**

To account for the limitations described in C.1., we developed a new approach, as describes in detail in the main text. It is based on a sliding window approach, which has been shown to be robust and reliable in identifying weather cues for masting (Journé, Simmonds, et al., 2025) and is commonly used through the R package *climwin* (Bailey & van de Pol, 2016) for many biological response variables (e.g., egg laying dates in birds). As *climwin* currently cannot fit zero-inflated negative binomial models, which are needed to accurately capture the structure of our dataset, we developed our own approach (for a general description see methods 3 main text).

Our sliding window approach tested a total of 15343 windows for each climate variable, and this was repeated four times to make sure that climate windows were stable and not selected randomly. For each window a zero-inflated negative binomial GLMM was fitted using *glmmTMB* (Brooks et al., 2017), with total annual seed count per tree as the response variable, temperatures in T1, T2 and T0 and precipitation in T1, T2 and T0, as well as total number of nuts of the previous year as explanatory variables and tree ID as random effect. The zero-inflated model was dependent on all fixed effects. For each set of 15343 tested windows, the focal climate variable could vary according to the window tested, while all other variables were kept constant to a certain window (i.e., first to the value of the window defined by the literature in the base model, then to the value of their best window of the previous iteration). Once a best window was defined for the focal variable by choosing the window with the lowest AIC, this variable was set to the value of this best window, and the next climate variable was tested. Hence, after the first iteration, all climate variables were set to their respective best windows defined by the first iteration, and the same procedure was followed for the second, third, and fourth iteration. To determine which measure for temperature is best suited to explain seed production, we additionally tested all windows for maximum, minimum and mean temperatures in the first iteration for all three years (T1, T2, T0) and chose the temperature measure that had the best window with the lowest AIC to be further used in the remaining models. For better understandability of this process, see Table S1 showing the exact specifications of climate variables that have been used in the four iterations.

**Table S1 Model specifications of iterative process to find sensitivity windows for each climate variable.** Each model includes a temperature and precipitation variable for year T0 (year of seed fall), T1 (year prior to seed fall) and T2 (two years prior to seed fall). Each model is run for every window of a length of 7 to 140 days between March 21 and September 22, resulting in 15343 windows. The focal variable varies according to the respective window, while the remaining variables are kept constant to a specified window. The window for which each variable is kept constant is specified in brackets: “base” = window commonly defined in the literature (June & July for T1 and T2 and May to August for temperature T0, March to April for precipitation T0), “best win In” = best window found in n^th^ iteration (n can be first, second, third or fourth), and “test” indicating the focal variable that can vary (additionally bold). After model 3, 6 and 9 it was decided based on the lowest AIC value, which of the temperature variables is used (min, max or mean temperature) in further models (i.e., max temperature for T1 and T2, mean temperature for T0). For the second iteration, all variables are set to their respective best window found in the first iteration and each variable is tested again and the same process is used for the third and fourth iteration (i.e., setting all variables to the best window of the previous iteration). All models are zero-inflated negative binomial GLMMs with tree ID as a random effect, number of nuts of the previous year as fixed effect, and individual-level total annual number of beechnuts as response variable (see methods c) in main text).

| **First iteration (I1)** |
| --- |
| 1. meanTempT0 (*base*) + **maxTempT1 (*test*)** + maxTempT2 (*base*) + precT0 (*base*) + precT1 (*base*) + precT2 (*base*) |
| 1. meanTempT0 (*base*) + **minTempT1 (*test*)** + maxTempT2 (*base*) + precT0 (*base*) + precT1 (*base*) + precT2 (*base*) |
| 1. meanTempT0 (*base*) + **meanTempT1 (*test*)** + maxTempT2 (*base*) + precT0 (*base*) + precT1 (*base*) + precT2 (*base*) |
| 1. meanTempT0 (*base*) + maxTempT1 (*best win I1*) + **maxTempT2 (*test*)** + precT0 (*base*) + precT1 (*base*) + precT2 (*base*) |
| 1. meanTempT0 (*base*) + maxTempT1 (*best win I1*) + **minTempT2 (*test*)** + precT0 (*base*) + precT1 (*base*) + precT2 (*base*) |
| 1. meanTempT0 (*base*) + maxTempT1 (*best win I1*) + **meanTempT2 (*test*)** + precT0 (*base*) + precT1 (*base*) + precT2 (*base*) |
| 1. **meanTempT0 (*test*)** + maxTempT1 (*best win I1*) + maxTempT2 (*best win I1*) + precT0 (*base*) + precT1 (*base*) + precT2 (*base*) |
| 1. **maxTempT0 (*test*)** + maxTempT1 (*best win I1*) + maxTempT2 (*best win I1*) + precT0 (*base*) + precT1 (*base*) + precT2 (*base*) |
| 1. **minTempT0 (*test*)** + maxTempT1 (*best win I1*) + maxTempT2 (*best win I1*) + precT0 (*base*) + precT1 (*base*) + precT2 (*base*) |
| 1. meanTempT0 (*best win I1*) + maxTempT1 (*best win I1*) + maxTempT2 (*best win I1*) + precT0 (*base*) + **precT1 (*test*)** + precT2 (*base*) |
| 1. meanTempT0 (*best win I1*) + maxTempT1 (*best win I1*) + maxTempT2 (*best win I1*) + precT0 (*base*) + precT1 (*best win I1*) + **precT2 (*test*)** |
| 1. meanTempT0 (*best win I1*) + maxTempT1 (*best win I1*) + maxTempT2 (*best win I1*) + **precT0 (*test*)** + precT1 (*best win I1*) + precT2 (*best win I1*) |
| **Second iteration (I2)** |
| 1. meanTempT0 (*best win I1*) + **maxTempT1 (*test*)** + maxTempT2 (*best win I1*) + precT0 (*best win I1*) + precT1 (*best win I1*) + precT2 (*best win I1*) |
| 1. meanTempT0 (*best win I1*) + maxTempT1 (*best win I2*) + **maxTempT2 (*test*)** + precT0 (*best win I1*) + precT1 (*best win I1*) + precT2 (*best win I1*) |
| 1. **meanTempT0 (*test*)** + maxTempT1 (*best win I2*) + maxTempT2 (*best win I2*) + precT0 (*best win I1*) + precT1 (*best win I1*) + precT2 (*best win I1*) |
| 1. meanTempT0 (*best win I2*) + maxTempT1 (*best win I2*) + maxTempT2 (*best win I2*) + precT0 (*best win I1*) + **precT1 (*test*)** + precT2 (*best win I1*) |
| 1. meanTempT0 (*best win I2*) + maxTempT1 (*best win I2*) + maxTempT2 (*best win I2*) + precT0 (*best win I1*) + precT1 (*best win I2*) + **precT2 (*test*)** |
| 1. meanTempT0 (*best win I2*) + maxTempT1 (*best win I2*) + maxTempT2 (*best win I2*) + **precT0 (test)** + precT1 (*best win I2*) + precT2 (*best win I2*) |
| **Third iteration (I3)** |
| 1. meanTempT0 (*best win I2*) + **maxTempT1 (*test*)** + maxTempT2 (*best win I2*) + precT0 (*best win I2*) + precT1 (*best win I2*) + precT2 (*best win I2*) |
| 1. meanTempT0 (*best win I2*) + maxTempT1 (*best win I3*) + **maxTempT2 (*test*)** + precT0 (*best win I2*) + precT1 (*best win I2*) + precT2 (*best win I2*) |
| 1. **meanTempT0 (*test*)** + maxTempT1 (*best win I3*) + maxTempT2 (*best win I3*) + precT0 (*best win I2*) + precT1 (*best win I2*) + precT2 (*best win I2*) |
| 1. meanTempT0 (*best win I3*) + maxTempT1 (*best win I3*) + maxTempT2 (*best win I3*) + precT0 (*best win I2*) + **precT1 (*test*)** + precT2 (*best win I2*) |
| 1. meanTempT0 (*best win I3*) + maxTempT1 (*best win I3*) + maxTempT2 (*best win I3*) + precT0 (*best win I2*) + precT1 (*best win I3*) + **precT2 (*test*)** |
| 1. meanTempT0 (*best win I3*) + maxTempT1 (*best win I3*) + maxTempT2 (*best win I3*) + **precT0 (test)** + precT1 (*best win I3*) + precT2 (*best win I3*) |
| **Fourth iteration** |
| 1. meanTempT0 (*best win I3*) + **maxTempT1 (*test*)** + maxTempT2 (*best win I3*) + precT0 (*best win I3*) + precT1 (*best win I3*) + precT2 (*best win I3*) |
| 1. meanTempT0 (*best win I3*) + maxTempT1 (*best win I4*) + **maxTempT2 (*test*)** + precT0 (*best win I3*) + precT1 (*best win I3*) + precT2 (*best win I3*) |
| 1. **meanTempT0 (*test*)** + maxTempT1 (*best win I4*) + maxTempT2 (*best win I4*) + precT0 (*best win I3*) + precT1 (*best win I3*) + precT2 (*best win I3*) |
| 1. meanTempT0 (*best win I4*) + maxTempT1 (*best win I4*) + maxTempT2 (*best win I4*) + precT0 (*best win I3*) + **precT1 (*test*)** + precT2 (*best win I3*) |
| 1. meanTempT0 (*best win I4*) + maxTempT1 (*best win I4*) + maxTempT2 (*best win I4*) + precT0 (*best win I3*) + precT1 (*best win I4*) + **precT2 (*test*)** |
| 1. meanTempT0 (*best win I4*) + maxTempT1 (*best win I4*) + maxTempT2 (*best win I4*) + **precT0 (test)** + precT1 (*best win I4*) + precT2 (*best win I4*) |

As there was only little variation in the selected best windows between the third and fourth iteration, if any, we did not do further iterations. The best window for each climate variable in each iteration is shown in Table S2. Given the strong signal and clear trough in AIC values for all climate variables in the fourth iteration (Figure S3), we are certain that selected windows are not random.


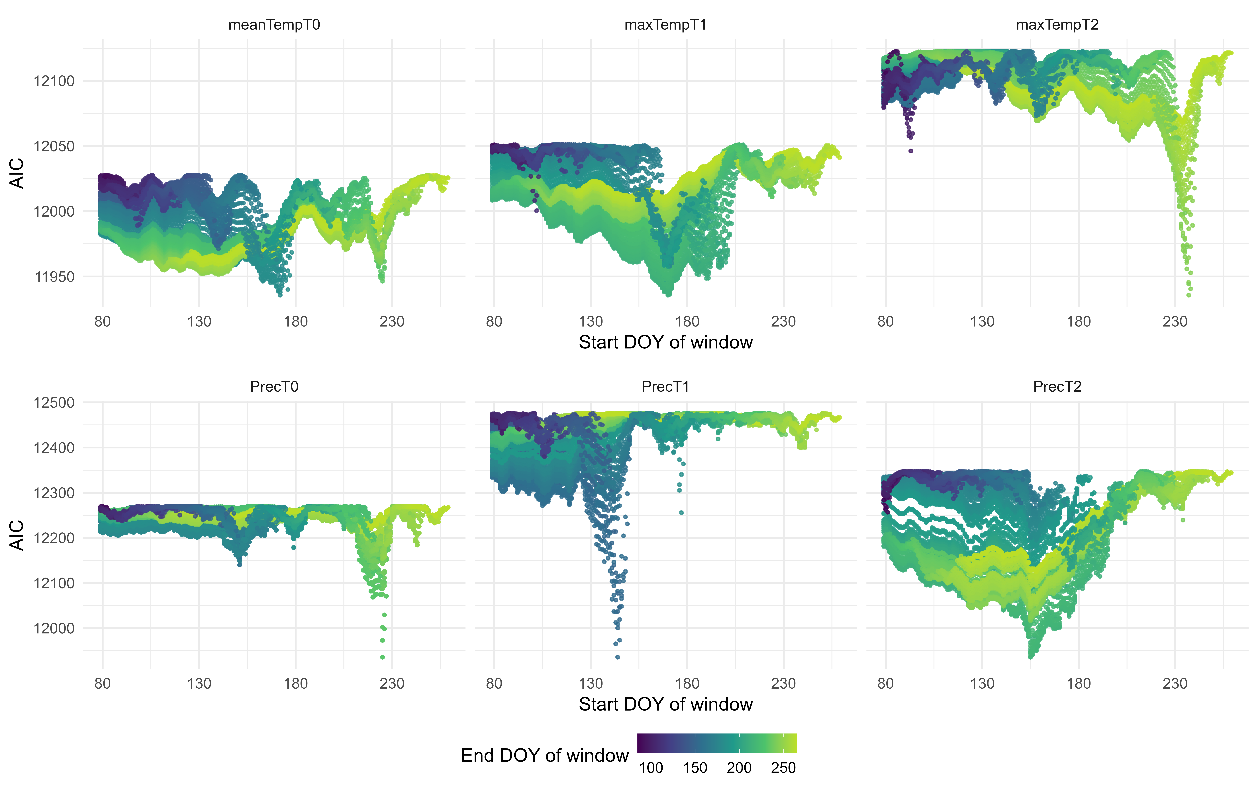
**Table S2 Best windows from all four iterations.** For each climate variable, the start and end days of the best window are given as DOY of the year and calendar date in parentheses (assuming the year was not a leap year), as well as the window length and the AIC of the model for all four iterations.

| Climate variable | Windows start | Window End | Windows length | AIC |
| --- | --- | --- | --- | --- |
| First iteration | | | | |
| Mean temperature T0 | 131 (12/05) | 245 (03/09) | 115 | 12473.12 |
| Max temperature T1 | 165 (15/06) | 204 (24/07) | 40 | 12588.18 |
| Max temperature T2 | 208 (28/07) | 215 (04/08) | 8 | 12511.33 |
| Precipitation T0 | 225 (14/08) | 231 (20/08) | 7 | 12163.27 |
| Precipitation T1 | 144 (25/05) | 154 (04/06) | 11 | 12297.49 |
| Precipitation T2 | 157 (07/06) | 253 (11/09) | 97 | 12248.7 |
| Second iteration | | | | |
| Mean temperature T0 | 176 (26/06) | 182 (02/07) | 7 | 12042.9 |
| Max temperature T1 | 171 (21/06) | 209 (29/07) | 39 | 12093.34 |
| Max temperature T2 | 237 (26/08) | 243 (01/09) | 7 | 12060.68 |
| Precipitation T0 | 225 (14/08) | 231 (20/08) | 7 | 11948.17 |
| Precipitation T1 | 144 (25/05) | 154 (04/06) | 11 | 12042.9 |
| Precipitation T2 | 155 (05/06) | 214 (03/08) | 60 | 11948.17 |
| Third iteration | | | | |
| Mean temperature T0 | 172 (22/06) | 181 (01/07) | 10 | 11936.9 |
| Max temperature T1 | 170 (20/06) | 212 (01/08) | 43 | 11947.28 |
| Max temperature T2 | 237 (26/08) | 244 (02/09) | 8 | 11937.62 |
| Precipitation T0 | 225 (14/08) | 231 (20/08) | 7 | 11936.47 |
| Precipitation T1 | 144 (25/05) | 154 (04/06) | 11 | 11936.9 |
| Precipitation T2 | 155 (05/06) | 215 (04/08) | 61 | 11936.47 |
| Fourth iteration | | | | |
| Mean temperature T0 | 172 (22/06) | 181 (01/07) | 10 | 11935.7 |
| Max temperature T1 | 170 (20/06) | 209 (29/07) | 40 | 11935.7 |
| Max temperature T2 | 237 (26/08) | 244 (02/09) | 8 | 11935.7 |
| Precipitation T0 | 225 (14/08) | 231 (20/08) | 7 | 11935.62 |
| Precipitation T1 | 144 (25/05) | 154 (04/06) | 11 | 11935.7 |
| Precipitation T2 | 155 (05/06) | 214 (03/08) | 60 | 11935.62 |

***Figure S3 AIC values of all tested windows per climate variable in the fourth iteration.*** *For each of the 15 343 windows tested per climate variable, the AIC value of the respective model is plotted against the start day of the window. For all variables, there is a clear pattern in AIC values with a through around a certain date visible. This indicates that the window with the lowest AIC, which is selected as the best window, is not selected by chance, as all windows around the same date with a similar length have low AIC values as well.*

**C.3. Comparison with approach used in Journé et al., (2024)**

Our new approach gives slightly different results than commonly reported in the literature when using established methodologies. To test whether the differences we find are based on biological or methodological differences, we additionally used the approach of Journé et al. (2024) on our data to assess whether we can replicate the results of Journé et al. for our data when using their approach, indicating a methodological difference, or still find different results, indicating a biological difference. We chose this study as a comparison, as their main finding, that temperature windows are anchored to the summer solstice, has broadly been picked up in following studies and because they use a large dataset covering several populations, rather than simulated data (Journé, Simmonds, et al., 2025), making it a good baseline to compare our findings to.


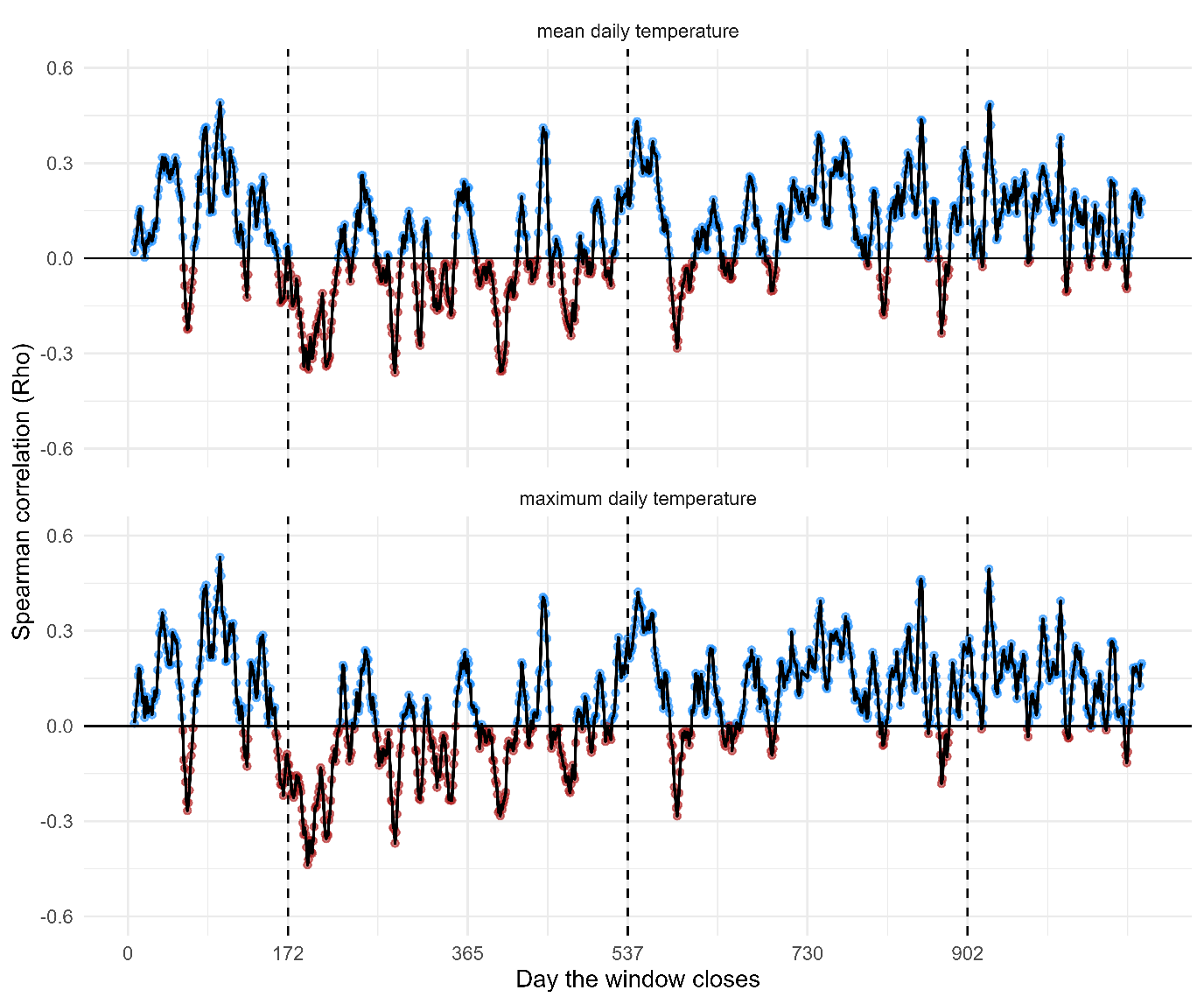


***Figure S4 Spearman correlation of log-transformed annual beechnut count and temperatures in a seven-day sliding window with one-day step-size.*** *The x-axis shows the day of the year on which the seven-day window closes, starting at January 1 of year T-2 (DOY 1) until December 31 of the year of seed fall (DOY 1095). All years are treated as non-leap years. The dashed lines indicate the day of the summer solstice in all three years. Blue dots show positive correlations, red negative correlations. The upper panel shows the correlation with mean daily temperatures (as used in Journé et al. (2024), the lower panel the correlation with maximum daily temperatures as used in the main analysis.*

Journé et al., (2024) also used a sliding window approach with a fixed window size of seven days and a one-day step size. For each window, they calculated Spearman rank correlations between the log-transformed mean total annual number of nuts per tree and the mean daily temperature over three years (year of seed fall and the two years before that). They found a clear peak in correlations for both years (negative for T2, positive for T1) opening at the summer solstice. As shown in Figure S4, we find a similar pattern, i.e., a negative peak in correlation coefficients starting at the summer solstice in T2 and a positive peak starting around the solstice in T1. In our population, these peaks are however not stronger than other peaks throughout the years, just slightly wider. For comparability with the results of our main approach, we additionally did the same analysis using maximum daily temperatures, resulting in neglectable differences compared to mean daily temperature (Figure S4, lower panel).

Given that Journé et al. report the means over many populations, the slight deviations in the results that we see for our populations are within an acceptable range, making our results (when using their methodology) comparable with theirs. This indicates that our study population, which has previously not been included in comparative analyses, is not an outlier, further suggesting that the differences in the identified cueing windows we find by using our new approach are due to methodological rather than biological differences. For further discussion see discussion of the main text.

**C.4. Collinearity test of explanatory climate variables**


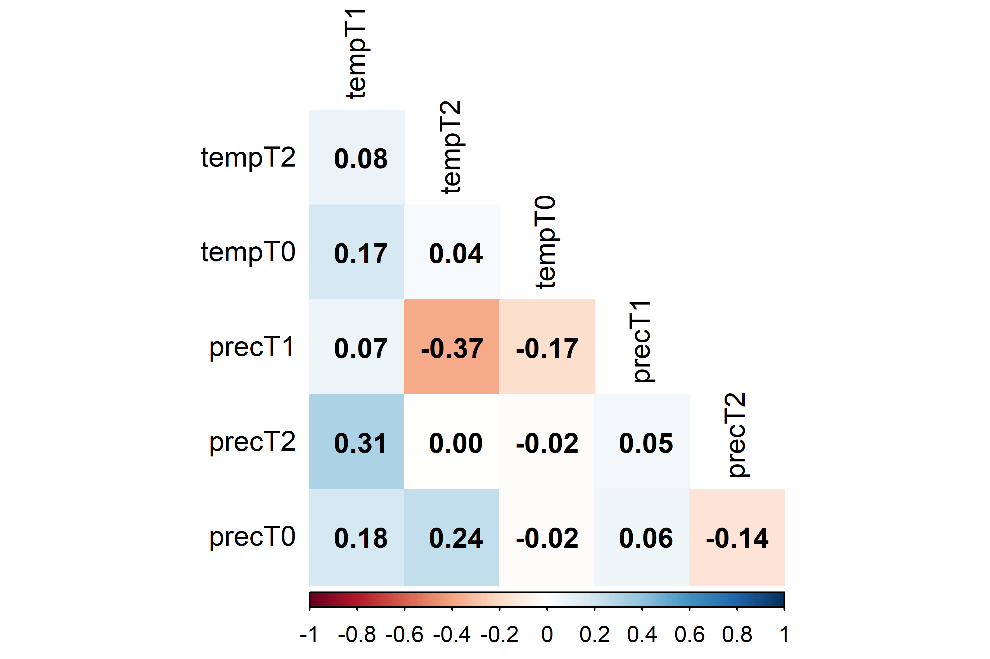
To exclude collinearity of our explanatory climatic variables, we calculated the Pearson correlation coefficient between each pair of climate variables, all set to their respective best window as determined in the fourth iteration (Figure S5). Correlation coefficients are low (i.e., absolute ρ < 0.4) for all variables, allowing us to fit all six variables into the final model.

***Figure S5 Correlation matrix of the six explanatory climate variables.*** *Numbers show the Pearson correlation coefficient (ρ) between each pair of climate variables. Colours facilitate the interpretation of these values, with darker red squares indicating more negative correlations, and darker blue squares more positive correlations. The correlation between climate variables is overall low, allowing to fit all variables into the same model.*

1. **Effect of internal resource reserves on seed production**

As the predictions of the final model could not accurately capture the masting breakdown and there was unexplained temporal variance remaining when fitting year back into the final model, climate variables likely interact with another factor, currently missing in the model. Since the internal resource budget of the tree determines its sensitivity to the climatic cues and ultimately determines how many resources can be allocated to reproduction, we additionally tested for the effect of individual resource availability on annual seed production. Following the method described in Kelly et al. (2025) and Rees et al. (2002), we calculated the resource reserves per individual tree based on its cumulative annual seed production throughout the study period. We then fitted resources of the previous year as an explanatory variable into the final model in an interaction with temperature in T1 for the conditional part of the model and as an additive effect for the zero-inflated part of the model^[[1]](#footnote-1)^, as we expected resources to be mainly related to the temperature cue in the year before seed fall, which is when the resources are used to form the flower primordia.

Resources did not show a significant effect on the number of nuts produced (interaction effect in conditional part: β = 6.041 * 10^-5^ ± 4.870 * 10^-5^, z = 1.24, p = 0.215) nor on the probability of reproducing (additive effect in zero-inflated part: β = 8.978 * 10^-5^ ± 1.764 * 10^-4^, z = 0.509, p = 0.611). When removing the interaction effect in the conditional model, resources did still not affect the number of nuts produced per tree per year (conditional part: β = 6.741 * 10^-5^± 4.730 * 10^-5^, z = 1.43, p = 0.154; zero-inflated part: β = 9.097 * 10^-5^ ± 1.767 * 10^-4^, z = 0.515, p = 0.607).

As previous studies (Kelly et al., (2025)) only included temperatures in T1 and T2 (one and two years prior to seed fall, respectively) in the model when testing for an effect of resources, we additionally also excluded temperatures in the year of seed fall and all three precipitation cues from the model. We again fitted an interaction of resources with temperatures in T1 for the conditional part of the model, and an additive effect for the zero-inflated part. In this model, resources still do not have an effect on the probability of a zero-year (β = 9.784 * 10^-5^ ± 1.203 * 10^-4^, z = 0.813, p = 0.416), while the interaction with temperature of T1 shows a significantly positive effect on the number of nuts produced, if there is reproduction (β = 1.671 * 10^-4^ ± 5.966 * 10^-5^, z = 2.80, p = 0.005). The temperature cue in T2 does then however no longer affect the number of nuts (β = -0.04428 ± 0.03670, z = -1.16, p = 0.247).

**References**

Bailey, L. D., & van de Pol, M. (2016). climwin: An R Toolbox for Climate Window Analysis. *PLOS ONE*, *11*(12). https://doi.org/10.1371/journal.pone.0167980

Brooks, M. E., Kristensen, K., Benthem, K. J., van, Magnusson, A., Berg, C. W., Nielsen, A., Skaug, H. J., Mächler, M., & Bolker, B. M. (2017). glmmTMB Balances Speed and Flexibility Among Packages for Zero-inflated Generalized Linear Mixed Modeling. *The R Journal*, *9*(2), 378. https://doi.org/10.32614/RJ-2017-066

Jantzen, C. C., & Visser, M. E. (2026). *Long-term annual seed production data of individual European beech (Fagus sylvatica) trees in the Netherlands*. ecoevorxiv. https://doi.org/https://doi.org/10.32942/X22T0H

Journé, V., Kelly, D., Hacket-Pain, A., Pearse, I. S., Szymkowiak, J., Foest, J. J., Kondrat, K., Oberklammer, I., Pesendorfer, M. B., Satake, A., & Bogdziewicz, M. (2025). Weather drivers of reproductive variability in perennial plants and their implications for climate change risks. *Nature Communications*, *16*(1), 9226. https://doi.org/10.1038/s41467-025-64300-6

Journé, V., Simmonds, E. G., Barczyk, M. K., & Bogdziewicz, M. (2025). Comparing statistical methods for detecting weather cues of mast seeding in European beech (Fagus sylvatica) across Europe. *Agricultural and Forest Meteorology*, *375*, 110857. https://doi.org/10.1016/j.agrformet.2025.110857

Journé, V., Szymkowiak, J., Foest, J., Hacket-Pain, A., Kelly, D., & Bogdziewicz, M. (2024). Summer solstice orchestrates the subcontinental-scale synchrony of mast seeding. *Nature Plants*, *10*(3), 367–373. https://doi.org/10.1038/s41477-024-01651-w

Kelly, D., Szymkowiak, J., Hacket‐Pain, A., & Bogdziewicz, M. (2025). Fine‐tuning mast seeding: As resources accumulate, plants become more sensitive to weather cues. *New Phytologist*, *246*(5), 1975–1985. https://doi.org/10.1111/nph.70092

Rees, M., Kelly, D., & Bjørnstad, O. N. (2002). Snow Tussocks, Chaos, and the Evolution of Mast Seeding. *The American Naturalist*, *160*(1), 44–59. https://doi.org/10.1086/340603

1. Model specification in R using *glmmTMB*: glmm(no. nuts ~ tempT0 + tempT1 * resources + tempT2 + precT0 + precT1 + precT2 + no. nutsT1 + (1| TreeID), ziformula = tempT0 + tempT1 + resources + tempT2 + precT0 + precT1 + precT2 + no. nutsT1 + (1| TreeID), family = nbinom2(), data = data) [↑](#footnote-ref-1)
